# Electrochemical discharge etching of porous tungsten emitters for field emission electric propulsion applications

**DOI:** 10.1038/s41598-026-53868-8

**Published:** 2026-08-12

**Authors:** Won Gyo Seo, Jung Won Kuk, Hongrae Kim, Dongsoo Kang, Jeongmoo Huh

**Affiliations:** 1 Division of Propulsion, Department of Small-Satellite Lab, Soletop Co., Ltd, Daejeon, 34051 Republic of Korea; 2 Satellite systems PMO, Advanced Defense Science & Technology Research Institute, Agency for Defense Development (ADD), Daejeon, 34186 Republic of Korea; 3 Department of Satellite Technology Development, Soletop Co., Ltd, Daejeon, 34051 Republic of Korea; 4 Department of Mechanical and Aerospace Engineering, College of Engineering, United Arab Emirates University, P.O. Box 15551, Abu Dhabi, UAE

**Keywords:** Porous emitter, Porous tungsten, Electrochemical discharge etching, Morphological transitions, Etching regimes, Field Emission Electric Propulsion, Energy science and technology, Engineering, Materials science

## Abstract

Field Emission Electric Propulsion (FEEP) systems offer high specific impulse and precise low thrust capability, making them suitable for micro propulsion applications. Porous tungsten is a promising material option for field emission emitters, offering advantages such as the formation of sharp tips and pore structures that enhance propellant transport through capillary action. However, the fabrication of well-defined porous tips remains insufficiently understood in terms of the relationship between etching process parameters and resulting structures. In this study, a spark-assisted electrochemical discharge etching was applied to fabricate porous tungsten emitter needles tailored for FEEP applications. Key process parameters, including applied voltage, power supply type, electrolyte type, molar concentration, and initial porosity, were investigated to establish an electrochemical etching domain. Distinct etching regimes associated with different morphological outcomes were identified, and the process window for forming viable porous emitter tips was found to be significantly constrained, with failure modes such as tip collapse, loss of porosity, and surface sealing observed outside this range. Under certain conditions, EDS analysis further revealed the formation of a multicomponent crust layer enriched in oxygen and potassium, which suppressed porosity preservation and influenced the resulting tip morphology. The fabricated emitter was integrated into a FEEP propulsion module, and preliminary field emission experiments conducted under vacuum conditions demonstrated emission feasibility.

## Introduction

Recent space missions involving miniaturised satellites, such as pico- and nanosatellites, have become increasingly sophisticated, demanding compact and efficient propulsion systems. For constellation and cluster orbit operations, downsized propulsion units are essential to enable precise attitude control and orbital manoeuvres. Among propulsion technologies applicable to miniaturised satellites, including Hall effect thrusters, resistojets, electrospray colloid propulsion, and field emission electric propulsion (FEEP), FEEP thrusters are particularly attractive for miniaturised satellites due to their high specific impulse and favourable thrust-to-power characteristics. Electric propulsion is becoming increasingly prevalent in satellites with masses below 50 kg^1^. In particular, electrospray-based systems, including FEEP, have emerged as a promising option for low power missions requiring high specific impulse and precise manoeuvring capability. FEEP systems employ electrostatic fields to accelerate charged particles and generate thrust. The fundamental electrohydrodynamic principles underlying field emission were first investigated in the early 20th century, and later formalised through the theoretical description of the Taylor cone^2^. Practical propulsion research based on these principles began in the late 1960s, initially using caesium as a propellant^3–5^, followed by the development of indium-based liquid metal ion sources (LMIS) as a safer alternative^6–10^.

As satellites became smaller and required thrust levels in the micronewton range, FEEP systems emerged as a suitable option for precision attitude control. Consequently, FEEP thrusters were considered for several space missions, including LISA (Laser Interferometer Space Antenna) Pathfinder^11^, GG (Galileo Galilei)^9^, and FFI (Free Flying Interferometer)^8^ projects of the European Space Agency (ESA). Although FEEP technology was evaluated during the development of LISA Pathfinder^12^, the mission ultimately adopted a micro cold gas system as the primary propulsion, while NASA’s ST-7 Disturbance Reduction System demonstrated colloid microthrusters^13^. Indium LMIS technology has been successfully applied for spacecraft charge control in ASPOC instruments across multiple missions^14^. More recently, Enpulsion has established significant flight heritage for operational indium-based FEEP thrusters since 2018, including long-duration operation and in-orbit extractor electrode self-cleaning^15^. Commercial FEEP systems have been developed, including those by Enpulsion based on technology from the Austrian Institute of Technology (AIT)^16^, and by Morpheus Space based on developments at the Technical University of Dresden, Germany^17^.

The FEEP propulsion system relies on electrostatic acceleration of ions generated at the emitter tip. Among its components, the emitter plays a critical role, as the electric field strength and resulting emission behaviour are strongly influenced by the tip geometry. As the emitter tip radius decreases, electrohydrodynamic emission can be achieved at relatively lower applied voltages, enabling efficient ion generation. Under sufficiently high electric fields, the liquid metal forms a conical structure, a Taylor cone, from which ion emission is initiated. The characteristics of propellant emission are strongly influenced by the electric field and emitter tip geometry. In particular, a smaller tip radius promotes ion emission, which is associated with higher specific impulse performance^18^. Therefore, the fabrication of sharp emitters is critical for efficient FEEP operation. In addition, the wettability and solubility between the emitter material and the propellant are important factors in emitter design. Sufficient wettability enables efficient propellant transport through capillary action, while low solubility helps maintain stable operation. Gallium and indium, which are widely used as FEEP propellants, exhibit good compatibility with tungsten in terms of both wettability and solubility.

Most studies on tungsten tip fabrication to date have focused on solid wires, largely motivated by applications in scanning probe microscopy (SPM) and electron or ion source technologies such as scanning tunnelling microscopy (STM) and focused ion beam (FIB) systems^19,20^. Consequently, the etching behaviours, geometric control strategies, and optimisation criteria reported in the literature predominantly reflect the requirements of solid, non-porous emitters, rather than the porous structures commonly employed in FEEP applications.

Porous tungsten is a promising emitter substrate for FEEP thrusters owing to its ability to enable passive capillary propellant feeding and its superior contamination resistance compared to solid tungsten. Tajmar et al.^21^ demonstrated a high-current indium liquid metal ion source based on porous tungsten crown emitters fabricated via micro powder injection moulding (µPIM), with individual needle tips sharpened to micron-scale apex radii using electrochemical etching. The 28-emitter array exhibited promising emission uniformity and operational stability. Vasiljevich et al.^22^ reported that porous tungsten needle emitters offer improved thermal cycling durability and contamination tolerance relative to solid tungsten needles. Further studies^23^ systematically compared emitter performance across substrates with varying grain sizes and sintering conditions. These investigations highlighted that the porous tungsten microstructure has a major influence on needle tip sharpness, which in turn affects mass efficiency and overall emitter performance for high specific impulse operation. Reissner et al.^24^ reported successful long-term endurance testing of a 28-needle porous tungsten crown emitter architecture without measurable performance degradation. This architecture has also been implemented in commercially available small satellite propulsion systems. Zhong et al.^25^ further characterised the relationship between emitter tip surface morphology and emission conductance using dynamic micro-electrochemical etching, demonstrating that fabrication techniques significantly influence field emission characteristics.

Collectively, these studies establish porous tungsten as a viable emitter material for indium FEEP thrusters, owing to its passive capillary feeding capability and superior contamination resistance compared to solid tungsten. However, a key limitation remains: electrochemical etching, a critical step required to sharpen emitter tips to micron-scale apex radii, has largely been treated as a preparatory, black-box technique rather than a subject of systematic investigation. In most cases, the etching process is described only briefly and qualitatively, with little to no detailed information on key process parameters such as applied voltage, electrolyte composition, current waveform, or substrate porosity. Failure modes specific to porous microstructures, including pore collapse, grain detachment, non-uniform dissolution, surface passivation, and product accumulation, are rarely discussed.

This gap has only recently begun to be addressed in the context of porous tungsten substrates. Sun et al.^26^ showed that conventional electrochemical etching protocols developed for solid tungsten are not directly transferable to porous structures, owing to the distinct electrochemical behaviour of interconnected pore networks and the significant accumulation of reaction products within the pores. As a result, additional process complexity is introduced, such as the need for interleaved ultrasonic cleaning cycles. Wang et al.^27^ advanced this by proposing a dynamic AC electrochemical etching method incorporating in-situ bubble-assisted cleaning enabled by AC excitation to mitigate product accumulation. Chen et al.^28^ also highlighted the strong coupling between microstructure engineering, particularly porosity and pore size control via powder metallurgy, and subsequent electrochemical sharpening processes for achieving stable high-performance emitters. However, these studies have primarily focused on specific process improvements or mitigation strategies under limited conditions, leaving the broader etching parameter space insufficiently explored.

Despite these recent advances, a comprehensive understanding of the electrochemical etching parameter space remains lacking. In particular, the regimes governing tip formation, morphological instability, structural failure, and the transition from electrochemical dissolution to discharge-dominated removal have not been systematically characterised.

The present study aims to address these limitations by systematically investigating the electrochemical etching behaviour of porous tungsten across a wide range of operating conditions. It identifies distinct operational regimes, ranging from controlled dissolution producing sharp emitter tips to spark-induced degradation and pore collapse, and establishes a process-level understanding of the etching behaviour. This work complements prior performance-oriented fabrication studies and recent mitigation-focused approaches, and defines an electrochemical etching domain through systematic classification of morphological transitions.

## FEEP system and micro-needle requirement analysis

Field Emission Electric Propulsion (FEEP) thrusters, owing to their low-thrust characteristics, are well suited for precise attitude control and drag compensation in satellite applications. In particular, the implementation of drag compensation, or drag-free control, in low Earth orbit (LEO) missions has been shown to improve image acquisition consistency, operational periodicity, and altitude accuracy for Earth observation satellites. For example, the use of three FEEP thrusters, each delivering 1 mN of thrust, can maintain the semi-major axis of a 100 kg class satellite within a deviation of approximately 3 m over a 24-hour period. These stringent mission requirements directly impose demanding constraints on the stability, controllability, and geometric precision of the emitter micro-needles used in FEEP systems.

To quantify the thrust requirements for long-term altitude maintenance, numerical analyses were conducted using FreeFlyer (AI Solutions) to simulate orbital altitude variations over a 365-day period under the influence of gravitational perturbations, solar radiation pressure, and atmospheric drag, both with and without active thruster operation. The simulations employed a Runge–Kutta integration scheme, while electrical energy consumption associated with attitude control and mission operations was not considered in this analysis. The input parameters used in the simulations are summarised in Table 1.

**Fig. 1:**
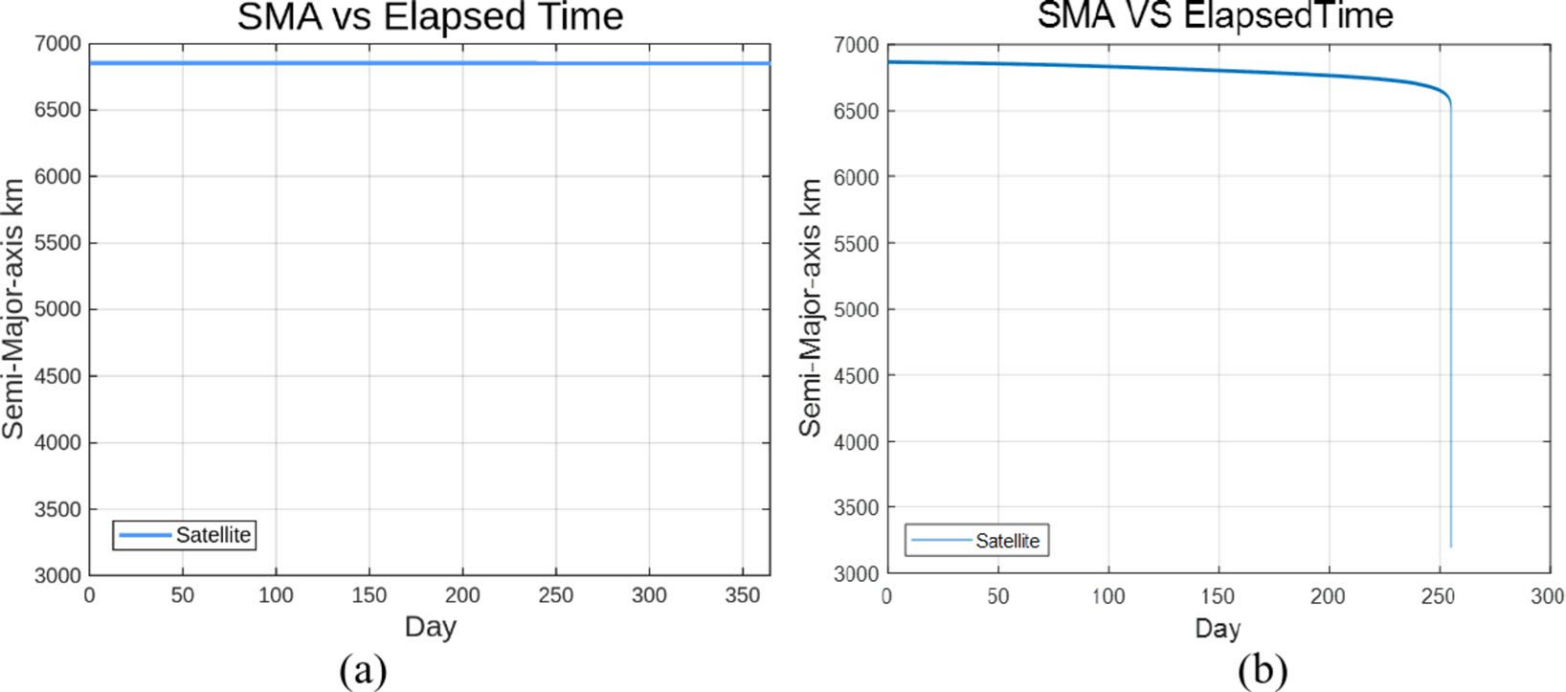
Time history of semi-major axis variation for a satellite (**a**) with active FEEP thrusting and (**b**) without thrust, illustrating the effectiveness of micro-thrust-based altitude maintenance.

For a 100 kg satellite equipped with three 1 mN class FEEP thrusters, continuous altitude maintenance over one year was achievable, as shown in Fig. 1(a). During this period, a total of 1,135 thruster firings were executed, corresponding to a propellant consumption of 0.31 kg. With approximately 15.2 orbital revolutions per day, thruster firings during eclipse and shadow periods were found to be justifiable. In contrast, without the use of thrusters, the satellite operational lifetime was predicted to be approximately 260 days, as shown in Fig. 1(b).

In this study, the target performance requirements for a FEEP thruster, consisting of multiple emitter needles powered by one power supply, were defined as a total thrust of 1 mN, a specific impulse exceeding 2000 s, and a power consumption not exceeding 80 W. Based on these requirements, the thrust generation characteristics of a single emitter needle were evaluated using Eqs. (1)–(4)^18,29,30^. Key performance parameters of a FEEP thruster include thrust (*T*), emission onset voltage (*V*_*0*_), emission current (*I*), and specific impulse (I_sp_). The emission onset voltage is determined using Eq. (1), which relates the distance between the extractor and emitter (*h*), emitter tip radius (*R*), surface tension (*γ*), and the vacuum permittivity (*ε*_*o*_), where the geometric parameters are defined as $\:{\eta\:}_{0}={(1+\mathrm{R}/\mathrm{h})}^{-1/2}$, $\:{\mathrm{r}}_{\mathrm{b}\mathrm{a}\mathrm{s}\mathrm{e}}$= R cosφ, and $\:{a=2h/\eta\:}_{0}$. The emission current (*I*) as a function of the applied voltage (*V*) is then calculated using Eq. (2), incorporating the onset voltage, atomic mass (*m*), elementary charge (*e*), and the Taylor cone half-angle (*φ*). Finally, the thrust and specific impulse are estimated using Eqs. (3) and (4), respectively, by introducing the beam divergence factor (*f*) and mass efficiency (*η*), which can be obtained experimentally or through analytical modelling.

1 $$\:{V}_{0}={\mathrm{t}\mathrm{a}\mathrm{n}\mathrm{h}}^{-1}\left({\eta\:}_{0}\right)\left(1-{\eta\:}_{0}^{2}\right)\sqrt{\frac{{a}^{2}\gamma\:}{{{\upvarepsilon\:}}_{0}{\mathrm{r}}_{\mathrm{b}\mathrm{a}\mathrm{s}\mathrm{e}}}}$$

2 $$\:\mathrm{I}=3\pi\:\sqrt{\frac{2e}{m}}\frac{\gamma\:Rcos\varphi\:}{2\sqrt{V}}\left({\left(\frac{V}{{V}_{0}}\right)}^{2}-1\right)$$

3 $$\:T=I\sqrt{2V\frac{m}{e}}\:f$$

4 $$\:{I}_{sp}=\frac{1}{g}\sqrt{2V\frac{e}{m}}\:f\eta\:$$

The emission current is typically limited to approximately 100 µA per emitter, as higher current levels increase droplet emission, reducing mass efficiency^31,32^, and accelerate erosion of the emitter tip^17,33^. Based on the estimated performance of a single emitter, the emitter tip radius and the number of emitters required to achieve a total thrust of 1 mN were determined to meet the defined performance requirements. The multi emitter performance was estimated by multiplying the single emitter thrust by the number of emitters, assuming independent emitter behaviour. The calculation assumed singly charged gallium ions, with an atomic mass of 69.723 u, corresponding to an ion mass of 1.157 × 10^− 25^ kg, and an elementary charge of 1.602 × 10^− 19^ C. A beam correction factor of 0.85 was employed to account for plume divergence, consistent with values reported in previous studies^16,34,35^. The distance of 2 mm between extractor and emitter was considered.

**Fig. 2:**
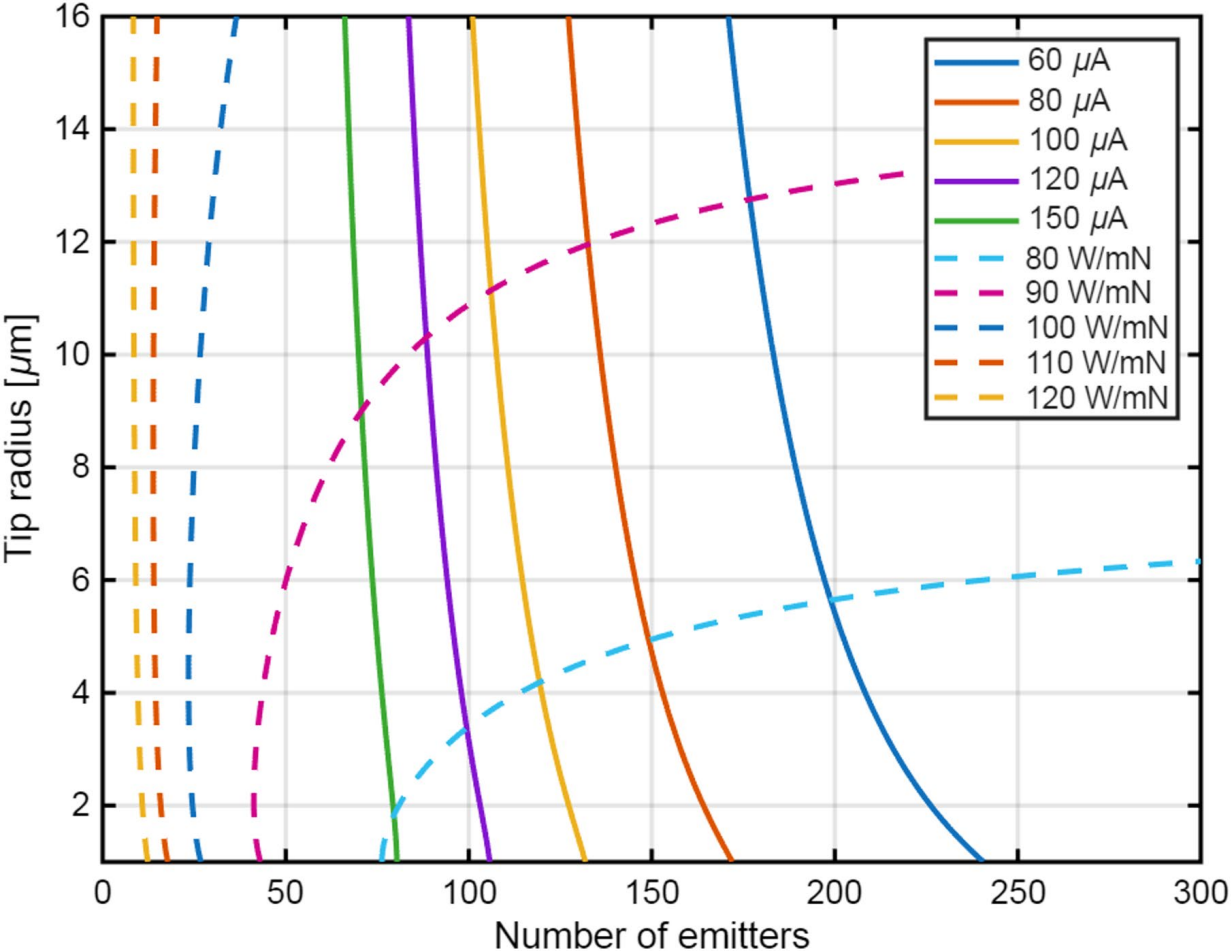
Required number of emitter needles and tip radius under various operating conditions of emission current and power-to-thrust ratio for a total thrust of 1 mN.

The performance was evaluated by systematically varying the emitter tip radius and applied voltage. It should be noted that the number of emitters is not an independent variable, but a derived quantity required to achieve the target total thrust, calculated based on the single emitter thrust corresponding to each operating condition. The relationships between emitter tip radius, number of emitters, emission current, and power-to-thrust ratio were evaluated simultaneously and are presented in Fig. 2. The solid lines represent constant single emitter current levels required to achieve a total thrust of 1 mN, while the dashed lines indicate constant power-to-thrust ratio conditions corresponding to the same total thrust. For a given emission current, a decrease in emitter tip radius leads to an increase in the required number of emitters to achieve the target total thrust. Conversely, larger tip radii reduce the required number of emitters but require higher operating voltages, which in turn affect the power-to-thrust ratio.

Under the constraints of a maximum power-to-thrust ratio of 80 W mN^− 1^ and a maximum emission current of 100 µA, the intersection of the corresponding limiting curves indicates a minimum of approximately 120 emitters, with an emitter tip radius of about 4 μm. For a 1 mN class FEEP thruster with a multi emitter array, an emitter tip radius in the range of 1–4 μm can be identified as a feasible design region, within which larger tip radii allow a reduced number of emitters, while smaller radii require a greater number of emitters. Accordingly, the emitter fabrication process was targeted to achieve tip sizes within this range. To provide a practical safety margin, a tip diameter smaller than 5 μm was set as the fabrication target during the etching process.

The identified tip radius range of 1–4 μm is consistent with values reported in previous studies on porous tungsten FEEP emitters. For instance, Tajmar^18^ and Vasiljevich et al.^16^ fabricated µPIM-based porous tungsten needles with tip radii of 2.25 μm and 1–2 μm, respectively. Similarly, Sun et al.^26^ and Wang et al.^27^ presented conical porous tips with apex radii of approximately 1.9 μm and below 5 μm using dynamic electrochemical etching techniques. These tip radii align well with the present design target and are generally associated with favourable emission characteristics, including reduced onset voltage and efficient operation in mN-class FEEP systems.

**Table 1:** Orbital input parameters used for altitude maintenance analysis of a satellite with and without FEEP thruster operation.

Parameter	without thrust	with thrust
Semi-major axis* (km)	6878.137	6878.137
Eccentricity	0.001	0.001
Inclination (deg)	90	90
Right ascension of ascending node (deg)	0	0
Argument of perigee (deg)	0	0
True anomaly (deg)	0	0
Dry mass (kg)	100	96.1
Drag area (m^2^)	10.8186	10.8186
Drag coefficient (C_D_)	0.6	0.6
Solar radiation pressure area (m^2^)	10.8186	10.8186
Thrust (mN)	0	3
Thrust coefficient (N)	0	0.003
Specific impulse (sec)	0	2000
Power (W)	0	300
Fuel density (kg/m^3^)	0	7310
Fuel mass (kg)	0	3.9

## Micro-needle electrochemical etching process

Preliminary etching tests were conducted on solid tungsten rods with a cylindrical, non-porous geometry to establish suitable process parameters for subsequent porous tungsten etching. In this process, a tungsten wire served as the anode in an alkaline electrolyte (KOH or NaOH), with a platinum counter electrode as the cathode. A direct current (DC) voltage (1–10 V) was applied to induce anodic dissolution of tungsten into tungstate ions (WO_4_^2−^), accompanied by hydrogen evolution at the cathode.

As etching progresses, the diameter of the tungsten wire decreases, forming a tapered neck region near the electrolyte interface that eventually leads to material drop-off and tip formation. The resulting tip geometry is influenced by process parameters such as electrolyte concentration, applied voltage, and immersion depth. Alternative approaches, such as inverted etching, involve immersing the material in an inert solution following drop-off. However, for porous tungsten, infiltration of such solutions into the porous structure can lead to non-uniform internal etching, making these approaches unsuitable for the present study.

The etching process consisted of two stages: preliminary etching and main etching. The preliminary etching stage was performed to remove the passivation layer on the tungsten surface, promoting uniform etching. A 1 M KOH solution was used, and the tungsten needle was immersed and etched for 10–20 s under either a 5 V DC or a 50 Hz AC power supply. The governing electrochemical reactions are given as follows:

Cathode:

$$\:6{H}_{2}O\:+6{e}^{-}\to\:\:3{H}_{2}\left(g\right)+6O{H}^{-}\:$$

Anode:

$$\:\mathrm{W}\left(\mathrm{s}\right)+8{OH}^{-}\to\:{W{O}_{4}}^{2-}+4{H}_{2}O+6{e}^{-}$$

Overall reaction:

$$\:\mathrm{W}\left(\mathrm{s}\right)+2{OH}^{-}+{2H}_{2}O\to\:{W{O}_{4}}^{2-}+3{H}_{2}\left(g\right)$$

**Fig. 3:**
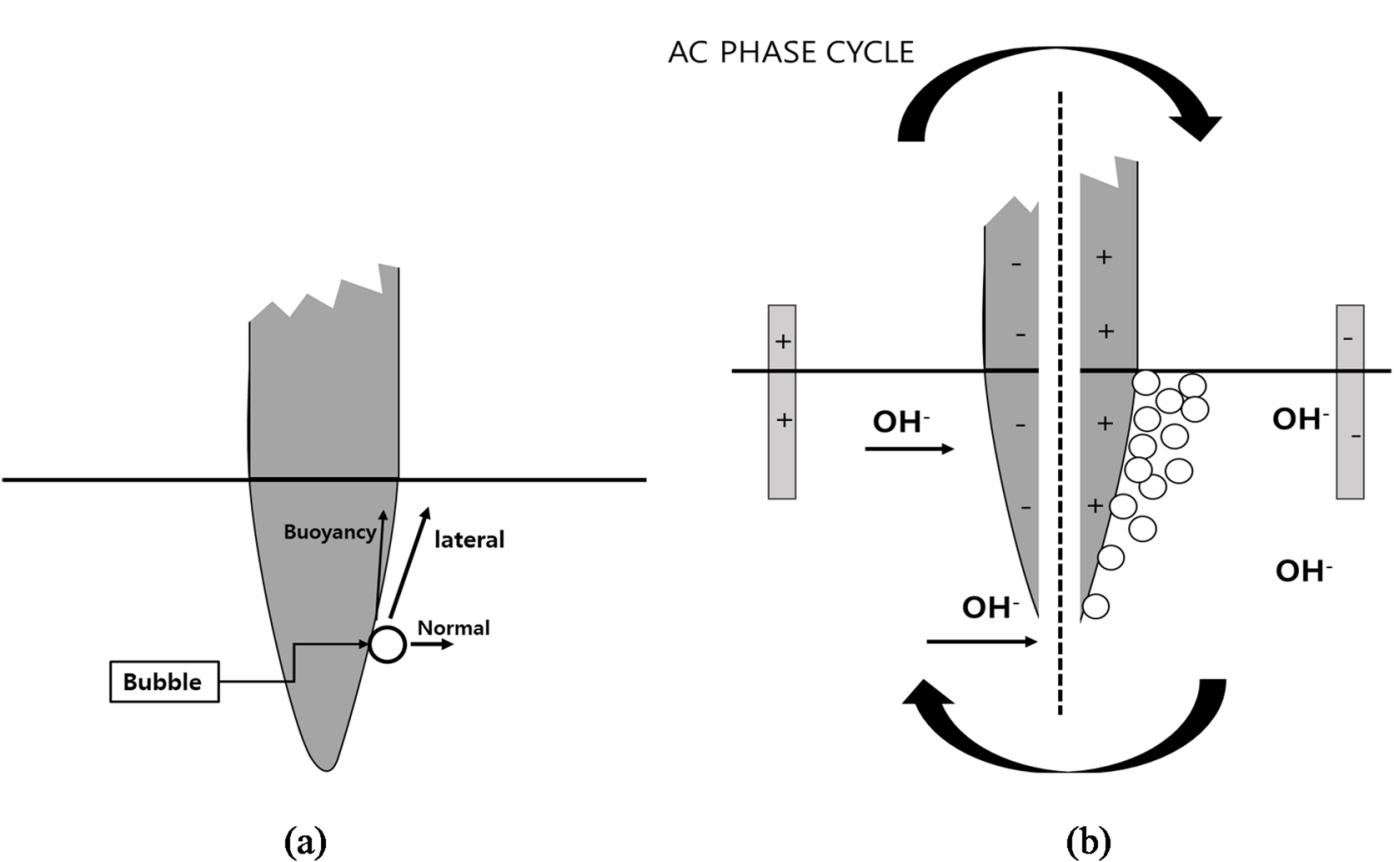
Force acting on gas bubbles during electrochemical etching (**a**), and reaction sequence under AC excitation illustrating transient bubble-induced shielding on the tungsten surface (**b**).

Aqueous KOH solutions with concentrations ranging from 1.0 to 11.4 M were prepared using ultrapure water. Electrolyte temperature is one of the parameters influencing the tip radius of etched tungsten needles, though its relative importance has been found to be lower than wire immersion depth and applied voltage^20^. Prior to each experiment, the solution was allowed to return to ambient conditions to minimise temperature variation between runs. The electrolyte temperature was maintained at approximately room temperature (23–25 °C) through passive cooling between etching steps. However, the lack of active temperature control represents a limitation of the present study, and improved temperature regulation would enhance reproducibility. The etched geometry of tungsten rods is governed by anodic dissolution in the presence of OH⁻ ions^36^, with local dissolution rates influenced by the electric field distribution and surface curvature. In addition, gas bubble formation on the tungsten surface affects the etching process, as illustrated in Fig. 3. Under AC excitation, gas bubbles are periodically generated at the electrode surface and may temporarily shield local regions from interaction with OH⁻ ions. However, periodic reversal of the electric field promotes bubble detachment, limiting their residence time on the surface. This dynamic bubble behaviour leads to variations in local etching rates, contributing to preferential material removal near the electrolyte interface and the formation of a sharpened tip geometry. During the main etching process, the immersion depth of the tungsten wire was carefully adjusted, and the applied voltage was gradually increased until material drop-off occurred, at which point the etching current ceased automatically. In addition to conventional electrochemical etching, a modified etching approach was developed in this study by introducing spark discharge phenomena at higher applied voltages, similar to those commonly observed in electrical discharge machining (EDM). This behaviour corresponds to an electrochemical discharge phenomenon, which has been mainly reported for insulating materials such as glass and ceramics. However, its application to metallic materials, particularly tungsten, has rarely been reported, and this approach was adopted to accommodate the unique characteristics of porous materials.

While similar approaches have been reported for micro-tip fabrication on platinum rods^37^, studies on porous tungsten remain limited. Existing research has primarily focused on minimising tip diameter, with less attention to the dependence of tip geometry on etching parameters. When conventional electrochemical etching is applied to porous tungsten, electrolyte infiltration via capillary action leads to internal etching within the emitter structure, making control of the etching process difficult^38^.

**Fig. 4:**
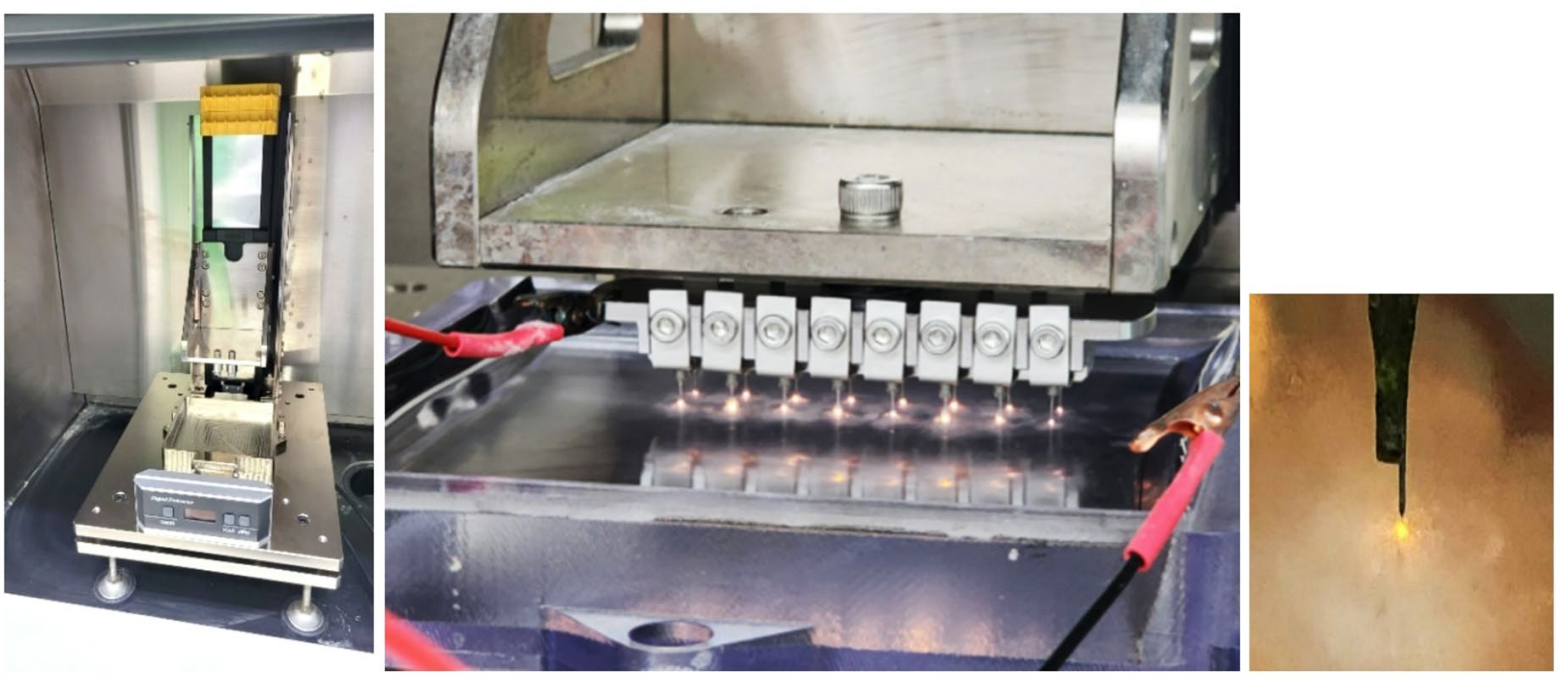
In-house-developed etching apparatus (left), simultaneous etching of an emitter tip array (middle), and single emitter tip etching showing electrochemical discharge phenomena during the etching process (right).

To mitigate this, the proposed approach incorporates discharge-assisted etching, enabling localised material removal while suppressing internal dissolution and promoting controlled shaping of the emitter surface. An appropriate voltage range was selected to induce both discharge-driven breakdown and electrochemical etching, enabling the formation of a sharp emitter tip in a single process. Etching was continued until the emitter tip diameter was sufficiently reduced, resulting in spontaneous material drop-off. All etching experiments were conducted using an in-house-developed apparatus, as shown in Fig. 4, where discharge-induced spark events were observed during the process.

## Electrochemical etching parameter study

Conventional electrochemical etching methods are not always well suited for the fabrication of porous tungsten micro-needles, owing to the intrinsic porosity of the material. The presence of interconnected pores increases the effective surface area available for electrochemical reactions, including reactions occurring within the internal pore network, which complicates the preservation of the original porous structure. During electrochemical etching, natural convection and gas evolution induce buoyancy-driven convective flows, including local vortex structures near the electrode surface, which generally facilitate reactant transport and removal of reaction products. However, in porous tungsten, the uneven surface topology and internal pore structure can disrupt these flows, leading to irregular bubble dynamics and spatially non-uniform etching behaviour.

Gas bubbles generated during electrochemical etching play a critical role in locally inhibiting the reaction by acting as a transient shielding layer between the electrolyte and the tungsten surface. Such bubble-induced shielding and its effect on local mass transport have been reported in gas evolving electrochemical systems, where bubble coverage can reduce the effective reaction area and influence species transport near the electrode surface^39,40^. When the buoyancy-driven convective flow near the electrode becomes non-uniform, this shielding effect also becomes spatially inconsistent, resulting in uneven surface protection. Such non-uniform surface protection alters the relative contribution of external surface etching and internal dissolution, potentially affecting the effective porosity of the material.

To suppress this undesired internal etching behaviour, the applied voltage range was extended beyond that typically used in conventional electrochemical etching to induce discharge phenomena similar to those employed in electrical discharge machining (EDM). By promoting discharge-assisted material removal, the contribution of purely chemical, convectively driven etching is reduced. As a result, discharge-induced erosion is expected to preferentially occur at the external surface of the porous tungsten, while mitigating excessive electrolyte-driven internal dissolution, contributing to improved preservation of the original porous structure. The experimental voltage range was defined by selecting the upper bound of voltages typically used in conventional electrochemical etching as the lower limit, and the lower bound of voltages commonly employed in electrical discharge machining (EDM) as the upper limit. This range was chosen to span the transition from purely electrochemical etching to discharge-assisted material removal.

The key design variables considered in this study include the electrolyte type and concentration, the power supply type, the applied voltage, and the porosity of the tungsten material. To systematically investigate the influence of these variables, a set of five parametric experimental cases was designed. In each case, one or two independent variables were selectively varied while all other parameters were maintained as control variables.

### Case 1

Influence of Power Supply and Electrolyte Type.

The first experimental set was designed to examine the influence of the power supply type and electrolyte composition on the etching profile. Two power sources were employed: constant direct current (DC) and 50 Hz alternating current (AC), which are known to generate distinct electric field characteristics during electrochemical etching. In parallel, two alkaline electrolytes, potassium hydroxide (KOH) and sodium hydroxide (NaOH), were selected for comparison, as both are commonly used for tungsten etching owing to their high conductivity and chemical reactivity. The mechanism underlying the difference between KOH and NaOH electrolytes is not fully established, but variations in bubble behaviour under alkaline conditions are known to influence bubble detachment and local mass transport near the electrode surface^39,41^. As the electrolyte type affects gas evolution characteristics and transport behaviour, both KOH and NaOH were considered independent variables in this study. For this experimental set, the electrolyte molarity was fixed at 11 M, the applied voltage was maintained at 2 V, the tungsten material was non-porous (0% porosity), and the etching time was set to 220 s with an immersion depth of 1 mm. All other parameters were held constant to isolate the effects of the power supply and electrolyte type. Each condition was repeated five times to assess reproducibility.

### Case 2

Effect of Applied Voltage and Electrolyte Type.

Building on the observations from Case 1, the second experimental set focused on the applied voltage as a primary design parameter under different electrolyte conditions. A voltage range of 5–40 V was selected to extend beyond that typically employed in conventional electrochemical etching, enabling the evaluation of voltage-dependent transitions in etching behaviour. For this case, the power supply was fixed to 50 Hz alternating current (AC) in order to minimise electrode degradation that may occur during prolonged direct current (DC) operation. Potassium hydroxide (KOH) and sodium hydroxide (NaOH) electrolytes with a molar concentration of 1 M were employed, and non-porous tungsten rods were used to eliminate the influence of internal pore structures. The etching duration was maintained at 220 s, which was sufficient to observe tip evolution and detachment. At lower applied voltages, etching proceeds more slowly and tends to produce longer taper profiles, whereas higher voltages accelerate material removal but may induce surface irregularities. This case was designed to identify an operating voltage range that enables controlled tip formation, while maintaining surface integrity and process stability.

### Case 3

Effect of Electrolyte Concentration.

Electrolyte molarity is another key parameter influencing ionic conductivity and electrochemical etching kinetics. In this case, the concentration of potassium hydroxide (KOH) was varied from 3 M to 10 M under a fixed 50 Hz AC power supply and a constant applied voltage of 2 V. All other process parameters were held constant, and the experiments were conducted using solid tungsten rods with 0% porosity to eliminate the influence of internal pore structures. Higher OH⁻ ion concentrations enhance electrochemical reaction rates by increasing ionic conductivity and reducing diffusion limitations, whereas excessively high molarity promotes vigorous gas evolution, which may disrupt local electric field uniformity and induce localised over-etching. In addition, increased bubble generation can alter buoyancy-driven convective flow patterns and transient shielding effects, particularly near the tip region. This experimental case was therefore designed to identify an electrolyte concentration range that balances etching behaviour with controlled tip geometry formation. The etching duration in this case was extended beyond 300 s, which was sufficient to reach the drop-off condition through progressive necking of the tungsten rod.

### Case 4

Role of Material Porosity.

To evaluate the feasibility of applying electrochemical etching to porous tungsten materials, Case 4 introduced material porosity, 0% and 15%, as the primary design variable. Two electrolyte types, potassium hydroxide (KOH) and sodium hydroxide (NaOH), were also considered. The power source was fixed to a 50 Hz alternating current (AC), while the electrolyte molarity was maintained at 11 M and the applied voltage at 45 V. Etching was continued until natural drop-off occurred. Unlike solid tungsten, porous materials introduce additional complexity due to internal surface exposure, which significantly increases the effective reactive area. This can enhance material removal within the bulk while simultaneously disturbing buoyancy-driven convective flow and gas bubble detachment along the external surface. Such effects may alter the balance between external surface etching and internal dissolution, potentially affecting the uniformity, structural integrity, and reproducibility of the resulting emitter tips. This case was designed to evaluate the influence of material porosity and electrolyte type on etching behaviour and tip morphology. The observations from this case informed the development of the discharge-assisted etching approach in Case 5.

### Case 5

Combined Effect of Porosity and High Voltage.

In the final test, the applied voltage range was extended to include values up to 100 V, which are comparable to those commonly employed in electrical discharge machining (EDM). This approach was intended to alter the relative contribution of the underlying etching mechanisms, promoting discharge-assisted material removal in addition to conventional electrochemical etching. The rationale was that, in porous tungsten, the initiation of localised spark discharges on the external surface may reduce the contribution of purely chemical etching within the porous network, contributing to improved preservation of the internal pore structure. Two voltage conditions were investigated: one below the discharge initiation threshold corresponding to the conventional electrochemical regime, and one within the discharge-active regime. In both cases, potassium hydroxide (KOH) electrolyte was employed. It is anticipated that, at higher voltages, bubble-induced shielding effects become less influential, and material removal is increasingly governed by dielectric breakdown and localised thermal effects associated with discharge events. Such energy input is expected to preferentially modify the external surface while maintaining the internal pore architecture.

**Table 2:** Summary of parameters used in the parametric etching study.

	**Independent Variables**				**Control Variables**
**CASE** **1**	Electrolyte: • KOH • NaOH		Power source: • AC • DC		• Porosity: 0% • Applied voltage: 2 V • Molarity: 11 M • Etching time: 220 s
**CASE** **2**	**Independent Variables**				**Control Variables**
	Electrolyte: • KOH • NaOH			Applied voltage: • 5–40 V	• Power source: AC, 50 Hz • Porosity: 0% • Molarity: 1 M • Etching time: 220 s
**CASE** **3**	**Independent Variable**				**Control Variables**
	Molarity: • 3–10 M				•Electrolyte: KOH •Applied voltage: 2 V •Power source: AC, 50 Hz •Porosity: 0% •Etching time: 325 s
**CASE** **4**	**Independent Variables**				**Control Variables**
	Porosity: • 0% • 15%	Electrolyte: • KOH • NaOH			• Molarity: 11 M • Applied voltage: 45 V • Power source: AC, 50 Hz • Etching time: until drop-off
**CASE** **5**	**Independent Variables**				**Control Variables**
	Porosity: • 0% • 15%	Applied voltage: • 5–100 V			•Electrolyte: KOH •Molarity: 1 M •Power source: AC, 50 Hz •Etching time: until drop-off

## Results and discussion

The effects of various independent variables on the tungsten tip morphology and dimensions are analysed, based on the experimental results obtained from the systematic parametric study described in the previous section. The effects of power supply type and electrolyte on the etched tungsten tip diameter were evaluated based on the results of Case 1 in Table 2.

**Fig. 5:**
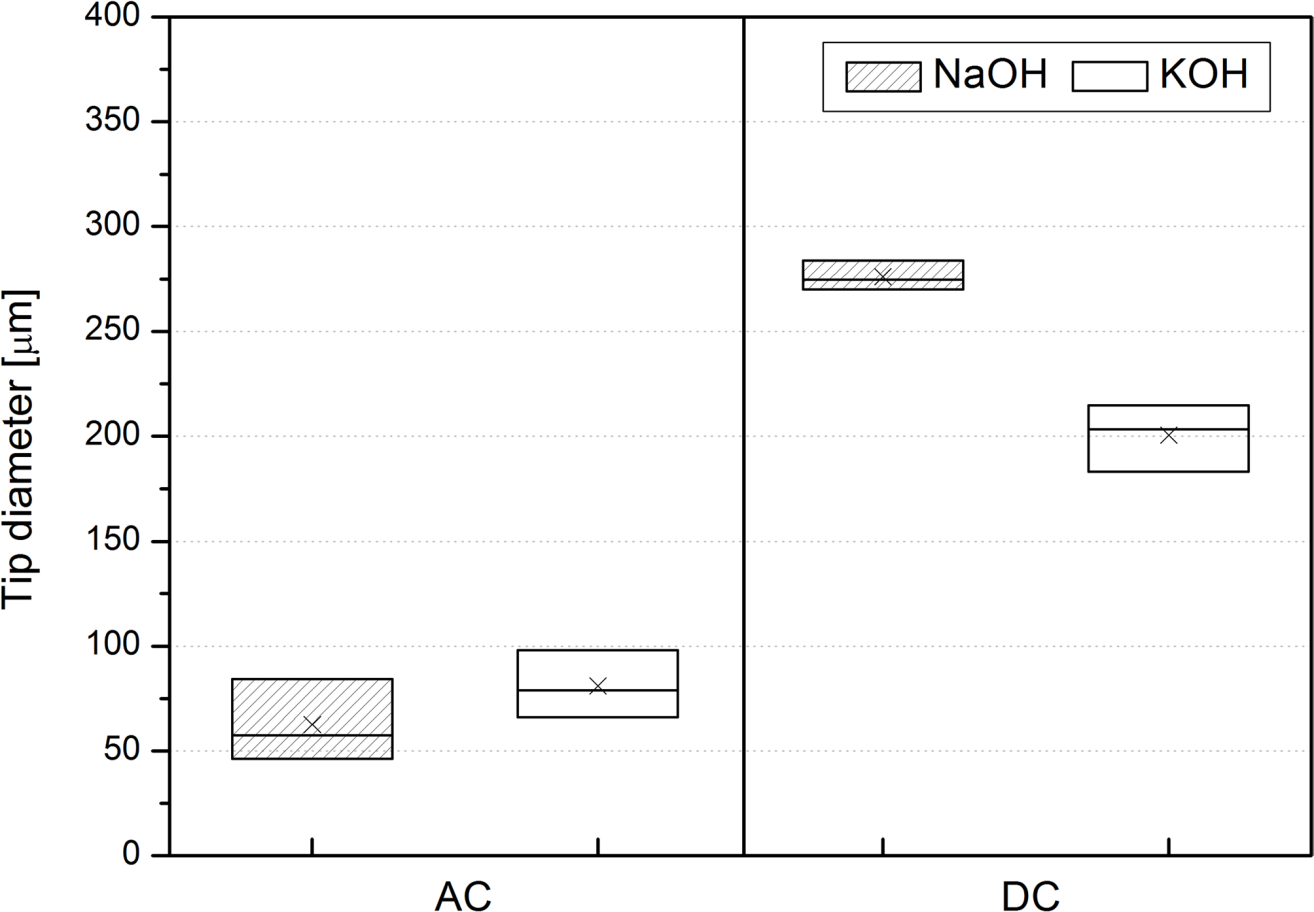
Achievable tip diameter range for solid tungsten needles under different power supply types and electrolyte solutions under Case 1 conditions (2 V, 11 M).

Figure 5 summarises the achievable tip diameter ranges for solid tungsten rods etched at an applied voltage of 2 V and an electrolyte concentration of 11 M using direct current (DC) and alternating current (AC) power supplies in potassium hydroxide (KOH) and sodium hydroxide (NaOH) solutions. Each condition was repeated five times, and the ranges represent the minimum and maximum values, and the crosses indicate mean values. Overall, AC power resulted in smaller etched tip diameters compared with DC power, irrespective of the electrolyte type. The influence of electrolyte composition was more pronounced under DC conditions, whereas its effect was relatively minor when AC power was used. The smallest tip diameters were obtained using AC power in NaOH solution, with values in the range of 46.3–84.2 μm. In comparison, AC etching in KOH yielded slightly larger tip diameters of 66.15–98.07 μm. In contrast, DC power consistently produced significantly larger tip diameters, ranging from 270 to 283.7 μm for NaOH and 183.3 to 214.7 μm for KOH. During the experiments, it was found that, under a direct current (DC) power supply, sustaining continuous electrochemical etching was difficult without external removal of the anodic product layer. The formation of a passivation film on the tungsten surface hindered the etching process and led to non-uniform taper formation. Based on these observations, the alternating current (AC) power supply was identified as more suitable for meeting the required fabrication specifications. Accordingly, all subsequent experiments were conducted using AC power as a controlled parameter.

**Fig. 6:**
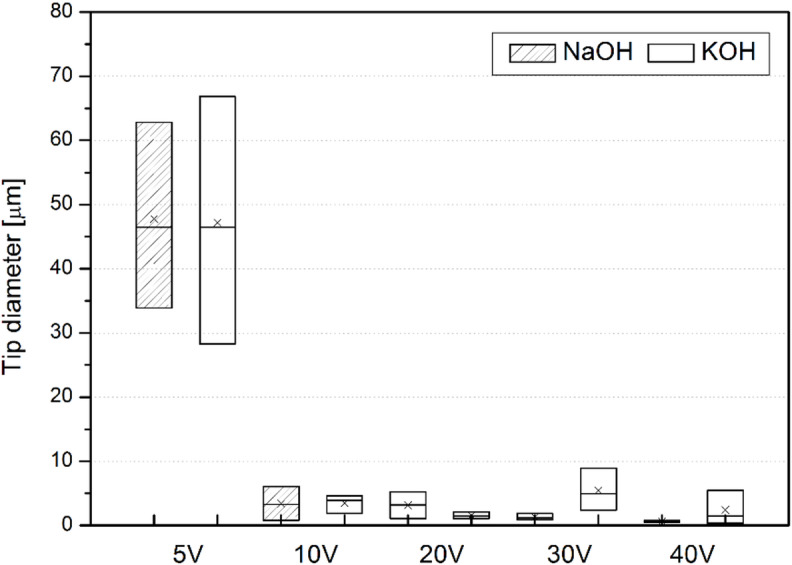
Achievable tip diameter range for solid tungsten needles as a function of applied voltage and electrolyte type under Case 2 conditions (50 Hz AC, 1 M).

Figure 6 shows the effect of applied AC voltage on the etched tip diameter for different electrolyte types (Case 2 in Table 2). For both potassium hydroxide (KOH) and sodium hydroxide (NaOH) electrolytes under 50 Hz AC power, the etched tip diameter exhibited a clear dependence on the applied voltage in the range of 5–40 V. In particular, a pronounced reduction in tip diameter was observed between 5 V and 10 V, indicating high sensitivity to voltage in the low-voltage regime. For NaOH electrolyte, the minimum tip diameters obtained at 5, 10, 20, 30, and 40 V were 33.90, 0.83, 1.08, 0.88, and 0.52 μm, respectively, while the corresponding values for KOH were 28.28, 1.89, 1.11, 2.41, and 0.32 μm.

**Fig. 7:**
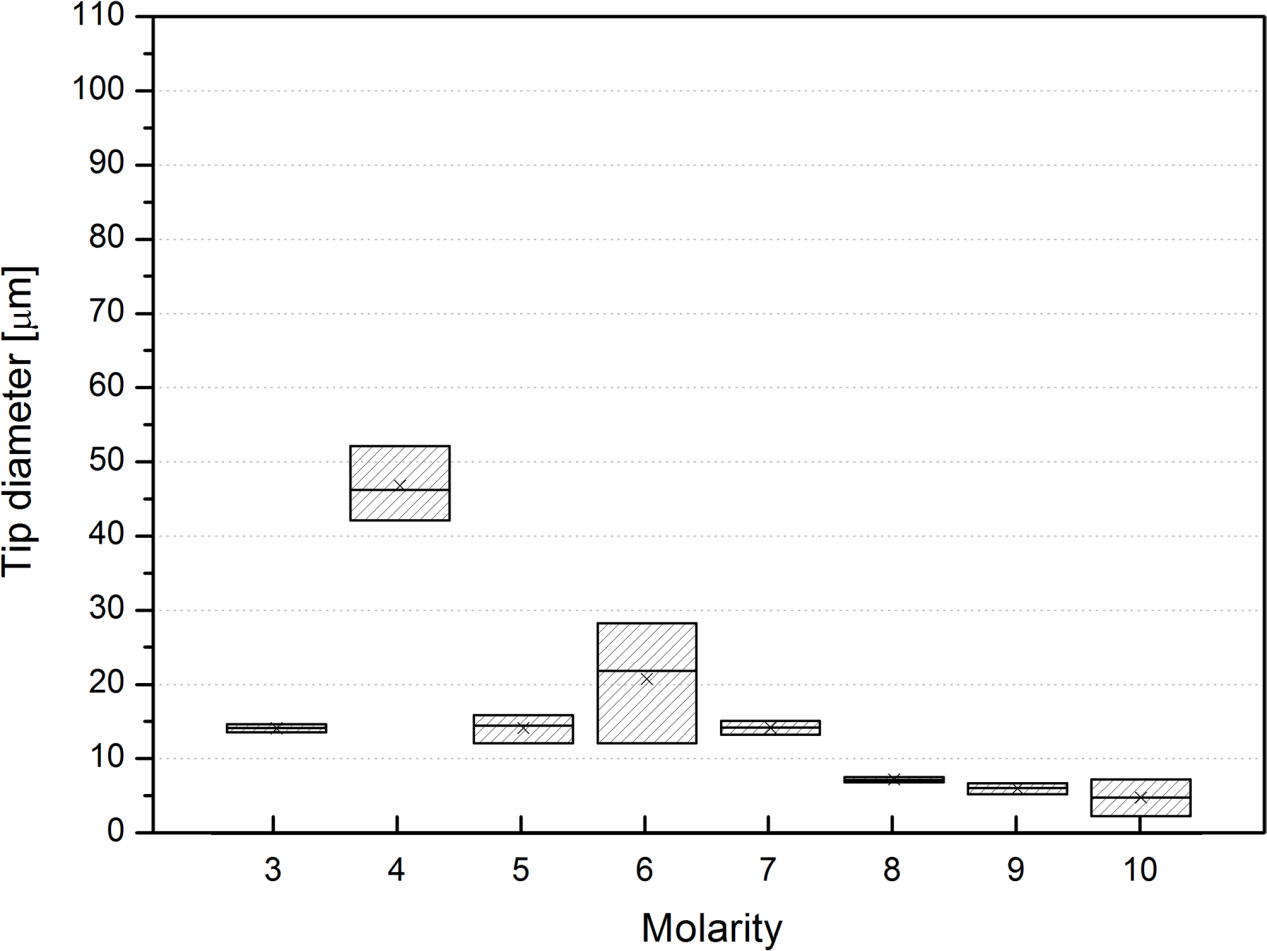
Achievable tip diameter range for solid tungsten needles as a function of KOH electrolyte molarity under Case 3 conditions (2 V, 50 Hz AC).

**Fig. 8:**
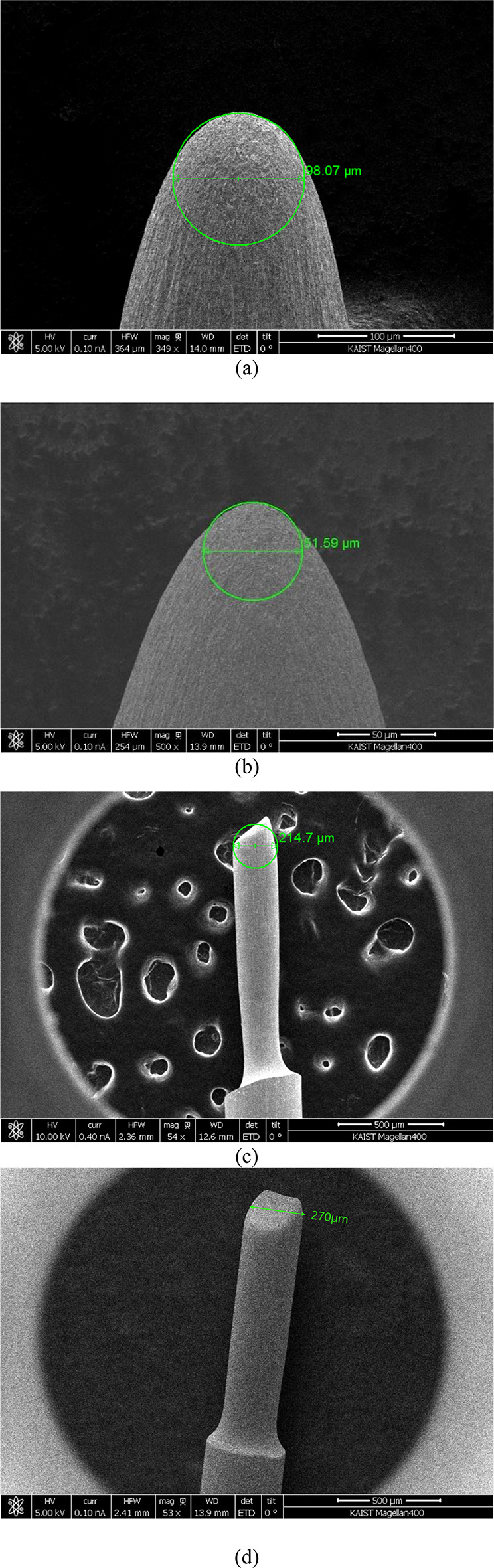
Representative SEM images of etched tungsten tip geometries fabricated under different power supply types and electrolyte solutions based on Case 1 conditions: (**a**) AC / KOH, (**b**) AC / NaOH, (**c**) DC / KOH, and (**d**) DC / NaOH.

The influence of electrolyte concentration on the etched tip diameter is shown in Fig. 7. The figure presents the achievable tip diameter range for solid tungsten rods etched using potassium hydroxide (KOH) electrolytes with molarities ranging from 3 M to 10 M under the conditions of Case 3 (2 V, 50 Hz AC). Under constant applied voltage and power supply, the etched tip diameter varied between 2.3 and 52.1 μm. Increasing electrolyte molarity enhanced ionic conductivity and electrochemical reaction rates, leading to accelerated material removal. However, at molarities above approximately 7 M, tip quality deteriorated due to excessive gas bubble generation, which disrupted local electric field uniformity and resulted in less controlled etching. The etching duration of up to 325 s was sufficient to observe the drop-off behaviour, with higher molarity conditions exhibiting earlier detachment events. These results highlight the need to balance electrolyte concentration to achieve efficient etching while maintaining controlled tip geometry. Representative scanning electron microscope (SEM) images of etched solid tungsten tips are shown in Fig. 8. In the images, the tips fabricated under AC power exhibited relatively smaller tip diameters (98.07 μm for KOH and 51.59 μm for NaOH), and showed smoother and more rounded apex profiles based on visual observation. In contrast, DC-powered etching produced significantly larger tip diameters (214.7 μm for KOH and 270 μm for NaOH), with apex geometries appearing sharper and surfaces exhibiting more irregular features.

**Fig. 9:**
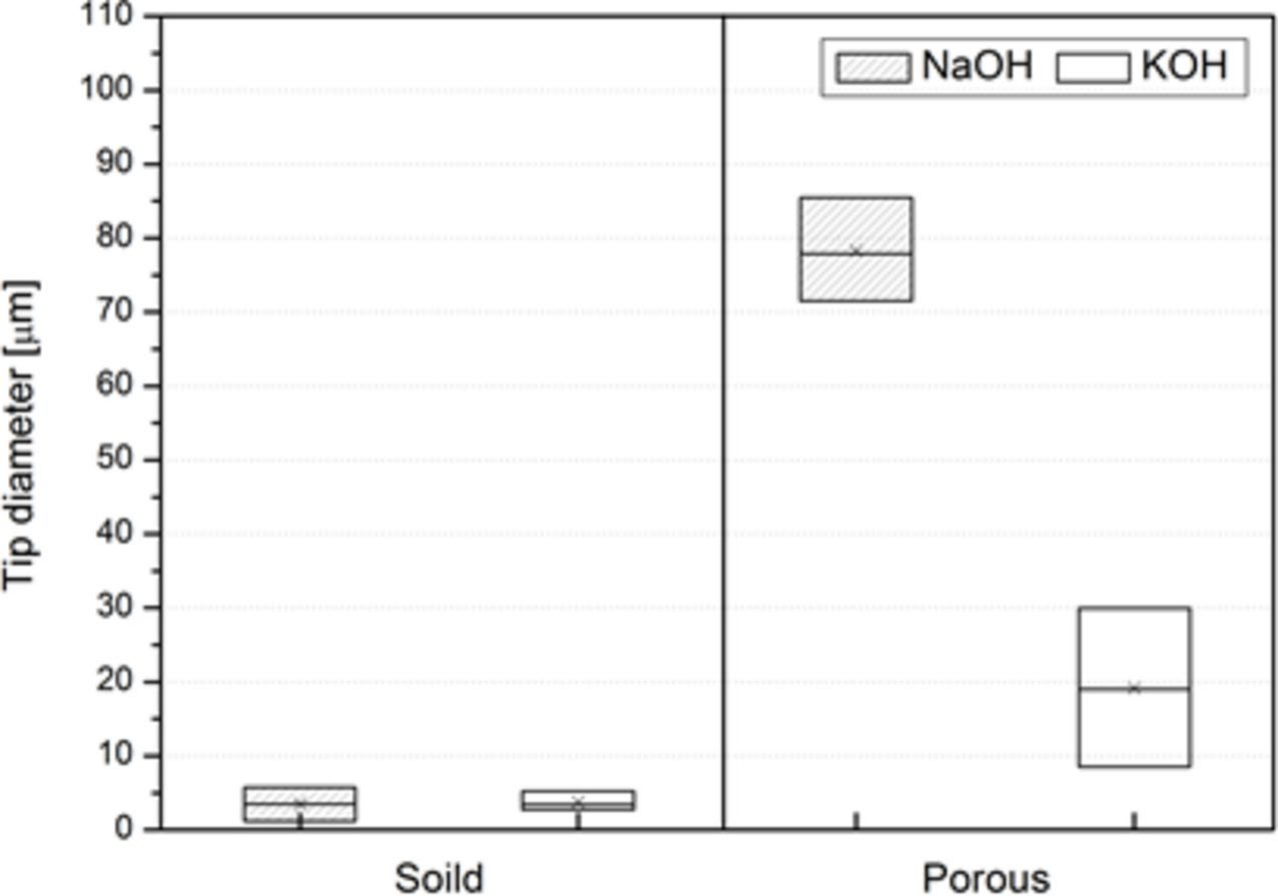
Comparison of achieved tip diameters for solid and porous tungsten needles etched using different electrolyte solutions (NaOH and KOH) under Case 4 conditions (45 V, 50 Hz AC, 11 M).

In order to examine the impact of material porosity on etching performance, solid and porous tungsten rods were etched under the conditions of Case 4. Figure 9 compares the etched tip diameters obtained for solid and porous tungsten needles processed at 45 V, 50 Hz AC, and an electrolyte concentration of 11 M using both NaOH and KOH solutions. The results show that solid tungsten needles were etched to an average tip diameter of 3.44 μm, which is significantly smaller than the average diameter of 48.45 μm obtained for porous tungsten needles. For solid tungsten, the difference in tip diameter between the two electrolytes was marginal. In contrast, a pronounced electrolyte dependence was observed for porous tungsten, with substantially smaller tip diameters achieved using KOH compared with NaOH. Specifically, the average tip diameter of porous tungsten etched in KOH was 19.05 μm, whereas that etched in NaOH was 77.85 μm. In addition to larger tip diameters, porous samples exhibited more irregular taper geometries than solid tungsten needles, based on visual inspection. This behaviour is likely associated with the increased internal surface area and more complex buoyancy-driven convective flow and bubble dynamics in porous structures, which may hinder uniform material removal during etching. This interpretation is consistent with previous studies on gas-evolving electrochemical systems, where bubble trapping and delayed detachment in confined or porous structures have been shown to influence local reaction environments and transport behaviour^40^.

**Fig. 10:**
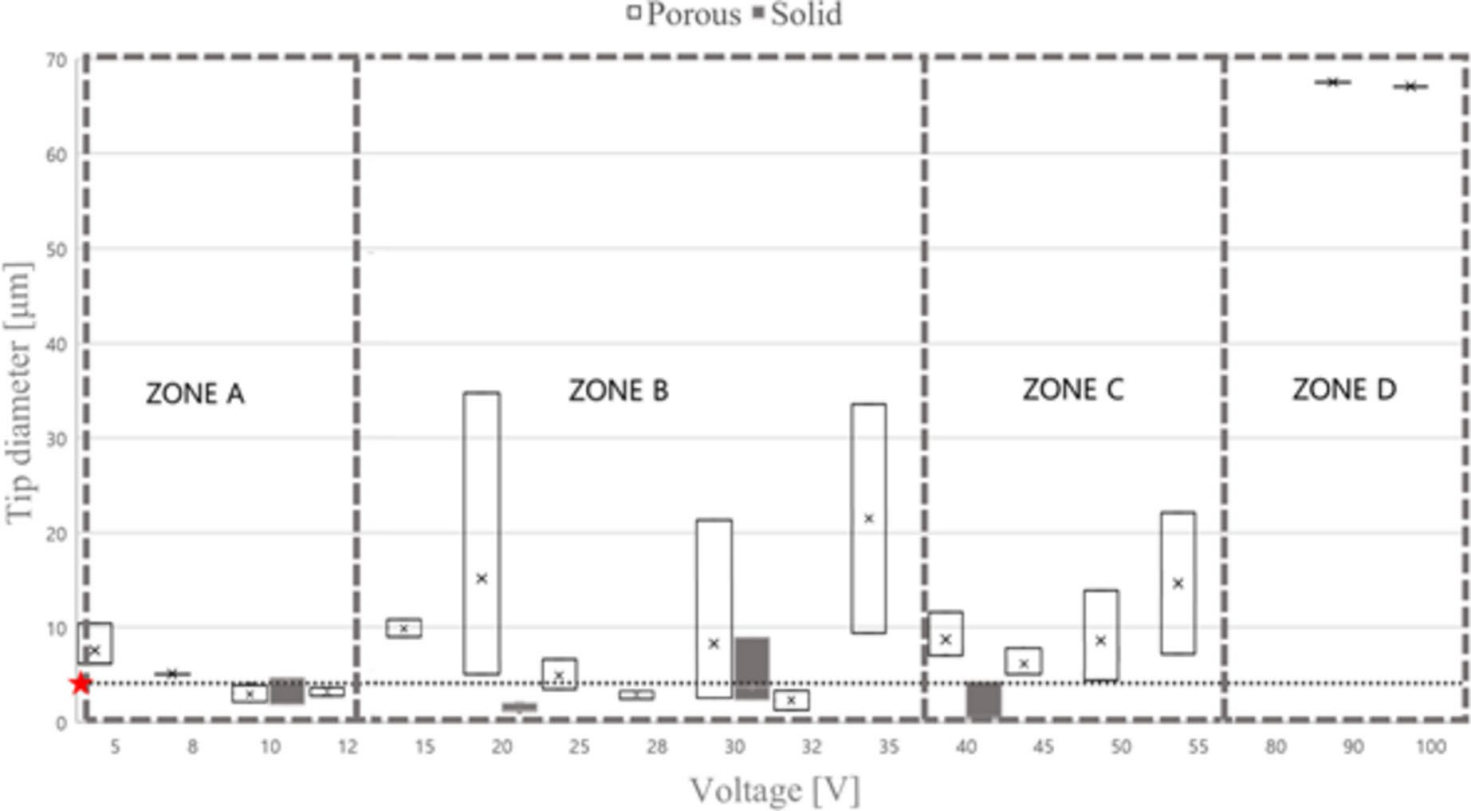
Achieved tip diameters for solid and porous tungsten needles as a function of applied voltage under Case 5 conditions (50 Hz AC, KOH, 1 M).

The combined effects of material porosity and applied voltage were investigated under the conditions of Case 5 (50 Hz AC, KOH, 1 M). As shown in Fig. 10, etched tip diameters for both solid and porous tungsten needles were evaluated over an extended voltage range up to 100 V. The resulting tip diameter exhibited a strong dependence on the applied voltage. Except for the highest voltage range of 80–100 V, later defined as Zone D, the target tip diameter of approximately 5 μm was generally achievable, although with varying success rates across different voltage regimes. Beyond tip diameter alone, distinct etching behaviours were observed as the applied voltage increased. In particular, higher voltages promoted discharge-assisted erosion, which limited deep chemical penetration into the porous structure and contributed to improved preservation of internal porosity. Based on the observed surface morphology and taper geometry, the etching results were classified into four voltage-dependent regimes, designated as Zones A–D.

ZONE A

**Fig. 11:**
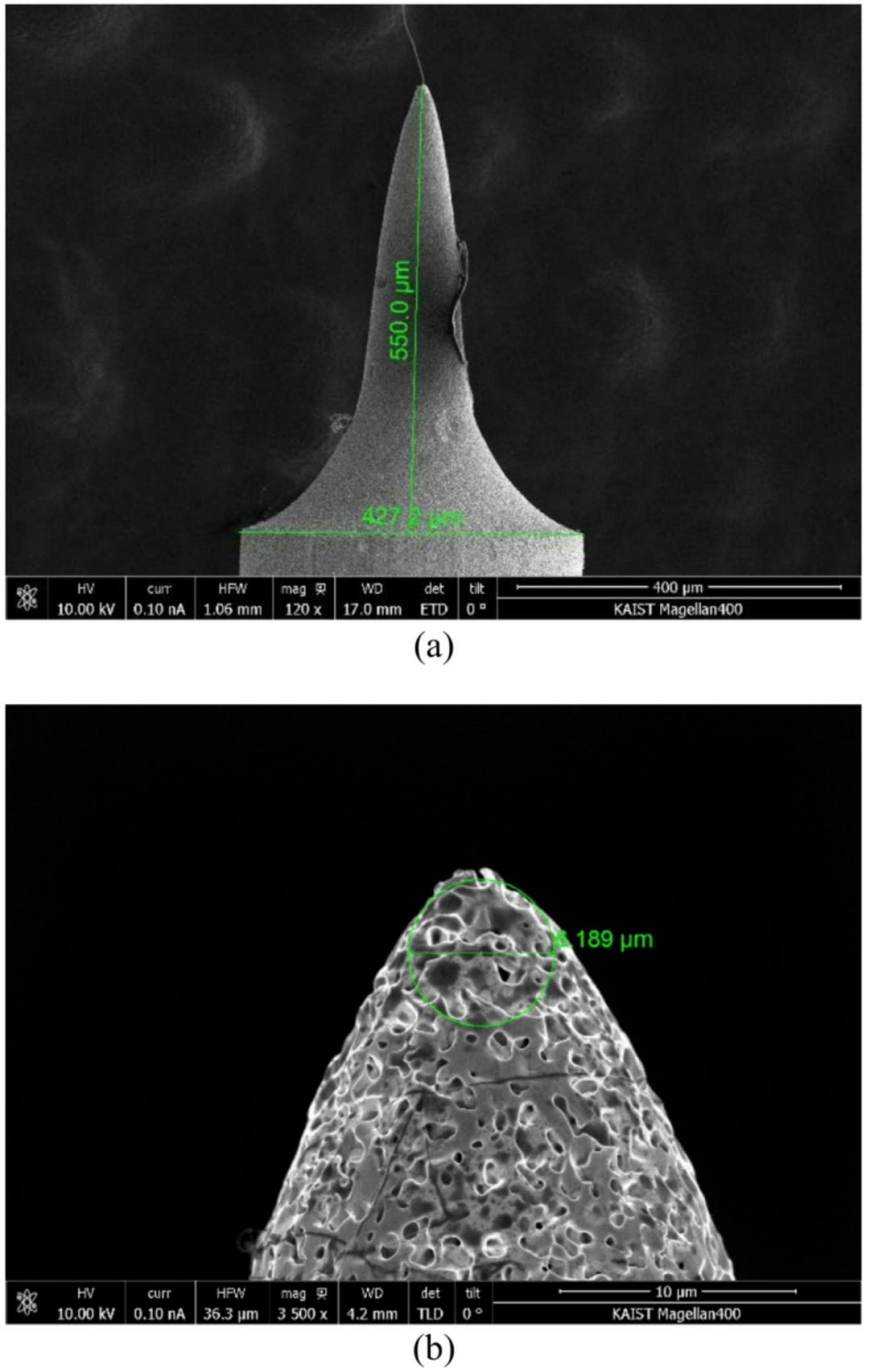
Etching results at 5 V corresponding to Zone A: (**a**) concave taper geometry of the needle, and (**b**) measured tip diameter.

In the relatively low voltage range from 5 V to below 12 V, both the tip diameter and overall porosity remained within the target range, as shown in Fig. 11(a). However, the etched needles consistently exhibited a concave surface profile, as illustrated in Fig. 11(b). Such concave geometries are generally unfavourable for FEEP emitter applications because they effectively reduce the load-bearing cross-sectional area near the emitter apex. This reduction increases local stress concentration under electrostatic loading. Given that the emitter tip is continuously subjected to erosion induced by ion emission during operation, elevated stress concentration is expected to accelerate geometric degradation, thereby reducing mechanical robustness and shortening operational lifetime. This behaviour may increase susceptibility to erosion-driven failure. From a capillary transport perspective, concave surface geometries are also less favourable than convex or bell-shaped profiles. Concave profiles can weaken capillary pressure gradients responsible for directing the propellant towards the emission site and may promote non-uniform wetting or local stagnation. Such effects are likely to compromise stable propellant replenishment during FEEP operation. Consequently, despite satisfying the geometric requirements in terms of tip diameter and porosity, needles fabricated in Zone A were deemed unsuitable due to their potentially inferior propellant delivery characteristics and the associated risk of reduced operational durability.

ZONE B

**Fig. 12:**
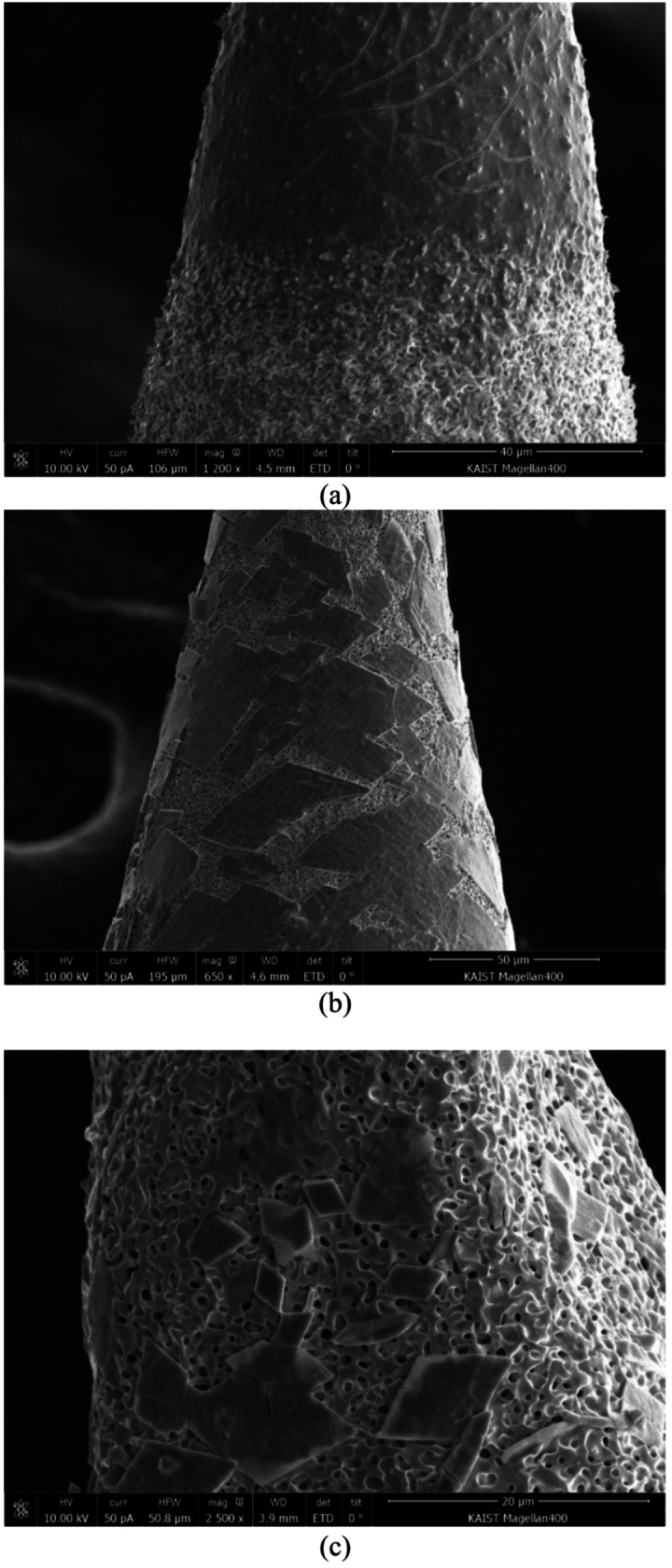
Evolution of surface morphology and porosity with applied voltage in Zone B: (**a**) surface crust formation at 12 V; (**b**) partial fracture of the crust revealing underlying porous structure at 32 V; and (**c**) increased exposure of the porous structure at 35 V.

In the intermediate voltage range of approximately 12–35 V, although the target tip diameter could be achieved in some cases, continued etching led to a progressive reduction in tungsten diameter, eventually triggering intermittent spark discharges. Compared with other voltage regimes, these discharge events persisted for longer durations, resulting in localised surface melting and the formation of a dense crust layer on the porous tungsten surface, as shown in Fig. 12. The development of this crust inhibited exposure of the underlying porous network and prevented the preservation of the desired porosity. As the applied voltage increased within this range, the surface morphology evolved noticeably. At 12 V, a continuous crust layer was observed on the emitter surface. At 32 V, partial fracture of the crust revealed portions of the underlying porous structure, while at 35 V, more extensive exposure of the porous surface occurred. This progressive transition indicates a competition between electrochemical dissolution, discharge-induced melting, and mechanical fracture of the surface layer. These observations demonstrate that, in this voltage regime, conventional electrochemical etching combined with insufficiently energetic discharge events can lead to surface sealing and porosity degradation in porous tungsten. This behaviour highlights the limitations of applying standard electrochemical etching conditions to porous materials without appropriate process adaptation.

**Fig. 13:**
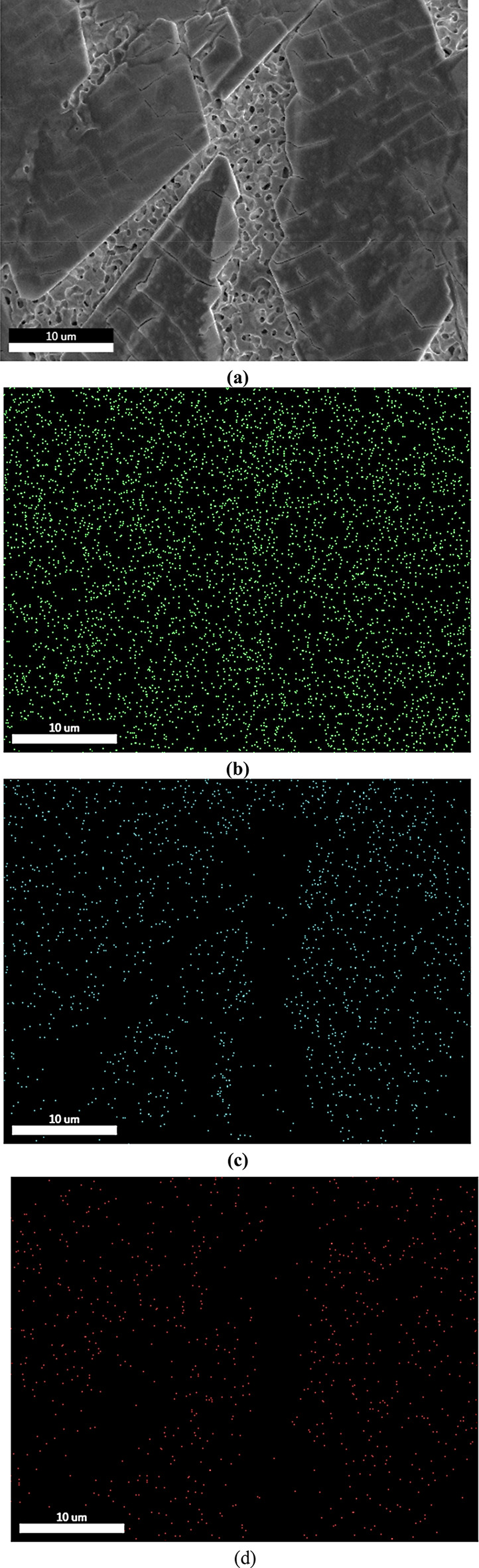
SEM image exhibiting the cracked plate-like morphology (**a**), and corresponding EDS elemental maps of tungsten (W) (**b**), oxygen (O) (**c**), and potassium (K) (**d**) in the analysed region.

To further examine the chemical nature of the crust layer, EDS analysis was performed on the surface region exhibiting the characteristic cracked plate-like morphology. The corresponding elemental maps of tungsten (W), oxygen (O), and potassium (K) are shown in Fig. 13. EDS elemental maps indicate that oxygen (O) and potassium (K) are relatively enriched within the cracked plate-like crust regions compared to the surrounding areas, while tungsten (W) is more uniformly distributed across the mapped field. Although the absolute signal intensities of O and K are moderate, their spatial co-localisation with the crust morphology is evident. Combined with the quantitative results (W 59.8 wt%, O 9.7 wt%, K 15.8 wt%) as listed in Table 3, these observations suggest that the crust is a multicomponent layer comprising tungsten-rich phases, oxygen-containing tungsten species, and K-bearing electrolyte-derived compounds, rather than a single phase of redeposited metallic tungsten or stoichiometric tungsten oxide (WO_3_).

ZONE C

The limitations observed in Zone B were mitigated by further increasing the applied voltage into the range of approximately 40–60 V. In this regime, the etching rate increased and discharge activity became more energetic but shorter in duration. As a result, surface melting observed at lower voltages was no longer sustained, and molten material was removed from the emitter surface. A peak current of up to approximately 40 A was measured at 50 V, corresponding to a transient power input of approximately 2 kW. The peak current occurred during significant fluctuations due to gas bubble formation and detachment at the electrode–electrolyte interface, which continuously altered the effective contact area. As the etching progressed, the effective contact area evolved, leading to a general reduction in current. Consequently, the process transitioned from discharge-dominated behaviour to electrochemical dissolution, and any discharge-induced surface irregularities were effectively removed during the etching process. In addition, the etching duration was short, typically less than 15 s compared to more than 1 min in Zone B. Together with continuous cooling by the electrolyte, this limited thermal accumulation and prevented severe damage to the emitter tip.

**Fig. 14:**
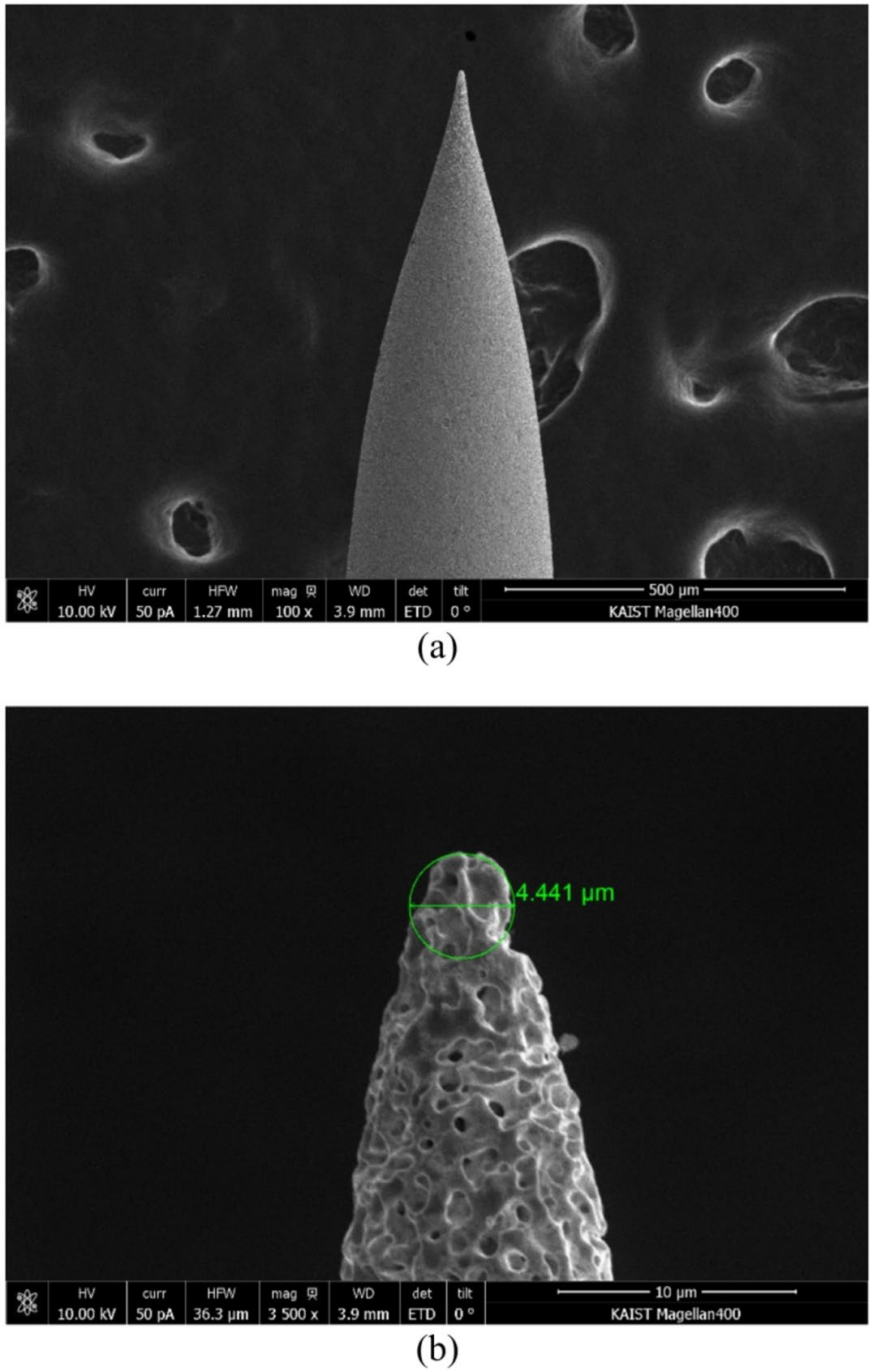
Etching results at 50 V corresponding to Zone C: (**a**) convex taper geometry of the needle, and (**b**) achieved tip diameter of 4.4 μm.

**Fig. 15:**
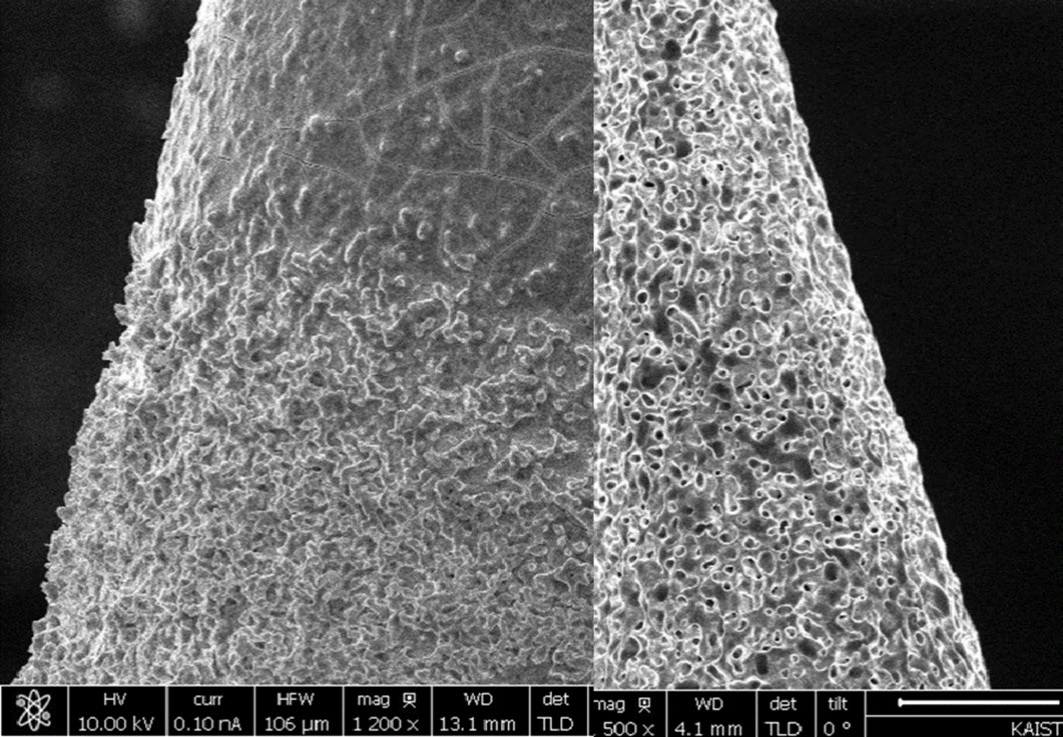
Comparison of fabricated porous tungsten emitters etched under different voltage regimes: loss of surface porosity due to crust formation at 20 V (Zone B, left), and preserved porous structure suitable for field emission at 45 V (Zone C, right).

Short duration, high energy discharge events promoted micro-fracturing of the surface layer, which effectively prevented the formation of a persistent crust and enabled continuous exposure of the underlying porous structure, as shown in Fig. 14. This discharge-assisted material removal mechanism differed fundamentally from the prolonged, low energy discharge behaviour observed in Zone B. In contrast to the concave surface profiles observed in Zone A (Fig. 11(a)), emitters fabricated under Zone C conditions exhibited a convex surface curvature. Such geometries are considered more favourable for stable propellant supply and emitter durability during field emission operation. A direct comparison between emitters etched at 20 V (Zone B), showing surface crust formation, and those etched at 45 V (Zone C), satisfying the porosity design requirement, is presented in Fig. 15. These results indicate that discharge-assisted etching within the Zone C voltage range provides a viable processing window for fabricating porous tungsten emitters with controlled geometry while preserving the porous structure. This regime was therefore identified as optimal among the investigated conditions.

ZONE D

**Fig. 16:**
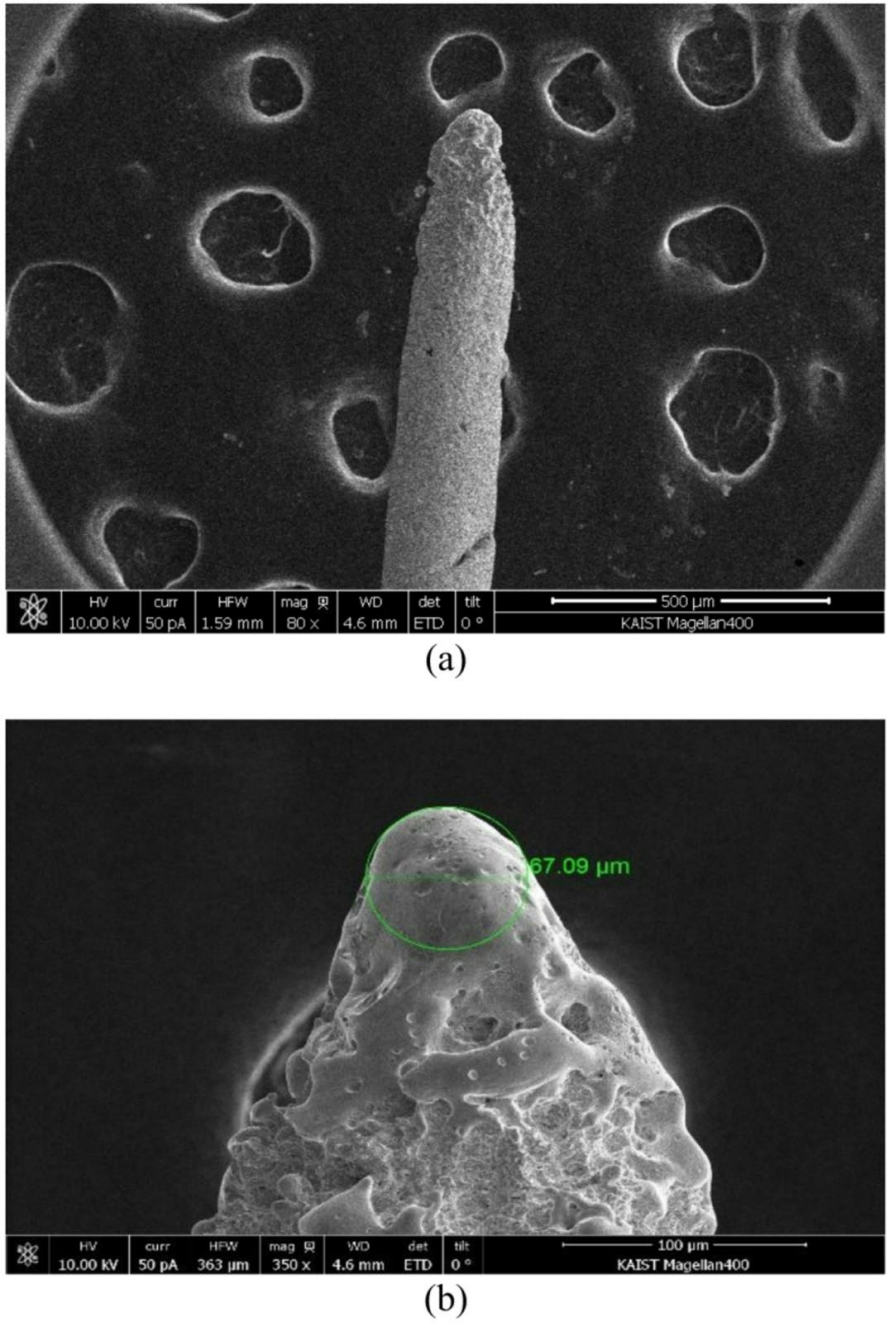
Morphological degradation of tungsten emitters at high applied voltages corresponding to Zone D: (**a**) 90 V, showing damage in the stem region near the tip; and (**b**) 100 V, showing surface melting at the tip.

At applied voltages higher than those defining Zone C, extending up to 100 V, the energy released during electrical discharge events became excessive relative to the energy required for controlled material removal. Under these conditions, discharge-induced heating dominated the process, leading to extensive melting of the porous tungsten material rather than progressive etching. In this high voltage regime, the balance between discharge-assisted surface erosion and electrochemical etching could no longer be maintained. As a result, both the emitter tip diameter and the internal porous structure deviated significantly from the target specifications. As illustrated in Fig. 16, severe surface melting and loss of porosity were observed, rendering this voltage range unsuitable for the fabrication of functional porous emitters.

These observations indicate that distinct etching behaviours occur across different applied voltage ranges in the electrochemical etching of porous tungsten. The morphological results were classified into four distinct zones (A–D), each corresponding to characteristic morphological transitions observed in SEM images, including concave taper (Zone A), crust formation (Zone B), convex porous tips with preserved porosity (Zone C), and excessive melting (Zone D). These results highlight that the underlying etching mechanism shifts with the applied voltage, from conventional electrochemical dissolution to discharge-assisted erosion, accompanied by various morphological transitions. When the applied voltage exceeds the discharge initiation threshold, discharge-assisted erosion becomes dominant, whereas excessive energy input leads to surface melting. Among the investigated conditions, the proposed discharge-assisted etching approach enables effective tip sharpening while preserving the internal porous structure within a defined voltage window.

**Fig. 17:**
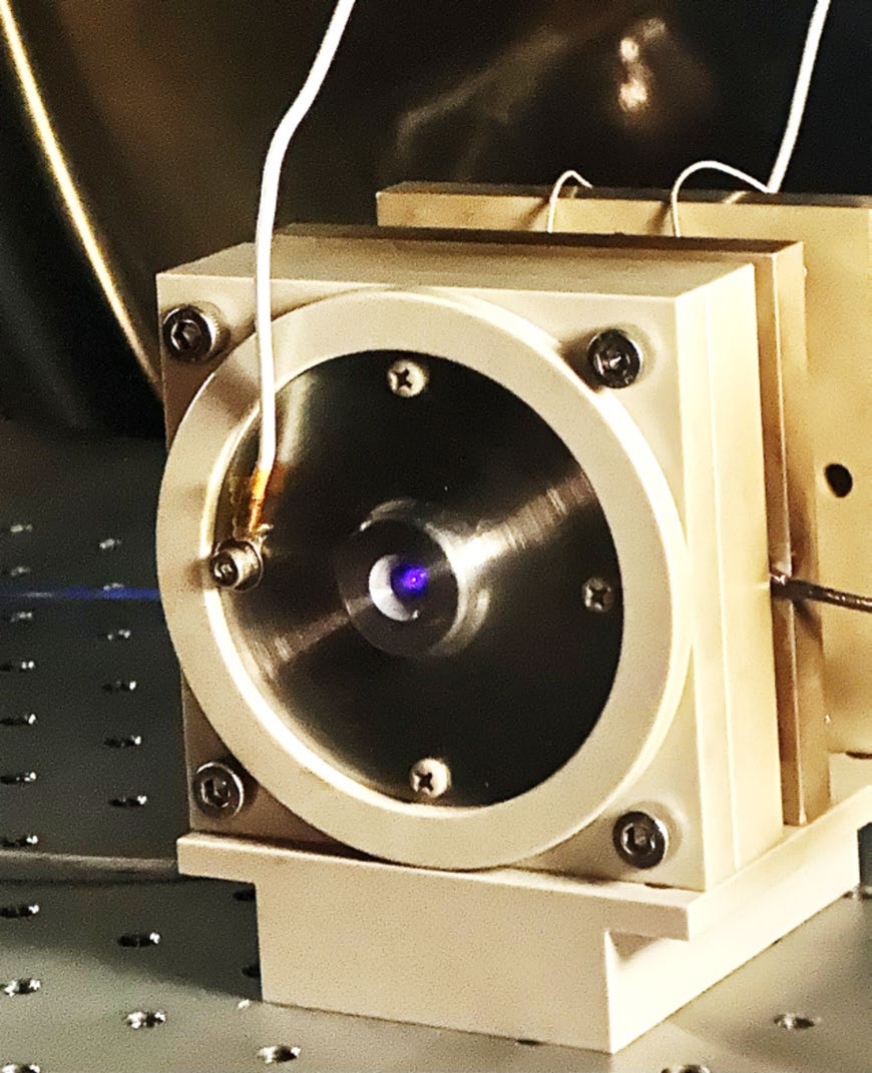
Ion beam emission observed during firing tests using a single porous tungsten emitter fabricated under Zone C conditions.

The porous needle fabricated using the proposed etching process was integrated into a FEEP thruster, and preliminary emission feasibility tests were conducted in a space-simulated vacuum chamber as shown in Fig. 17. Emission testing was conducted in a vacuum chamber made of SUS304 stainless steel with dimensions of Φ0.8 × 1.5 m, equipped with a rotary vane pump (WSA, W2V100) backed by a turbomolecular pump (OSAKA, TG1100F), achieving a base pressure of 1.6 × 10^− 6^ Torr (approximately 2.1 × 10^− 6^ mbar). The single porous tungsten emitter was mounted on a propellant reservoir and integrated into a FEEP module. The assembly was supported and electrically insulated using a Polyether Ether Ketone (PEEK) holder. The module comprised the emitter–reservoir assembly, a circular extractor electrode to establish a uniform electric field, and a resistive heater electrically isolated using alumina spacers. High voltage was supplied using a high-voltage power supply (SHV300R, Conver Tech, 0–30 kV, 10 mA). The voltage was automatically adjusted by the control system to maintain a target emission current of approximately 100 µA. The emission current and applied voltage were monitored through the power supply’s built-in measurement system, providing time-resolved measurements during operation.

Prior to emission testing, the porous tungsten emitter was wetted with liquid gallium propellant. Wetting was performed in a separate auxiliary vacuum chamber. A crucible containing high-purity gallium was heated using a Zero Voltage Switching (ZVS) induction heater under vacuum conditions. The emitter assembly was then immersed into the molten gallium, allowing capillary-driven infiltration into the interconnected pore structure. Performing the wetting process under vacuum conditions minimises oxide formation on both the liquid metal and tungsten surface, which would otherwise increase the contact angle and inhibit effective infiltration.

**Fig. 18:**
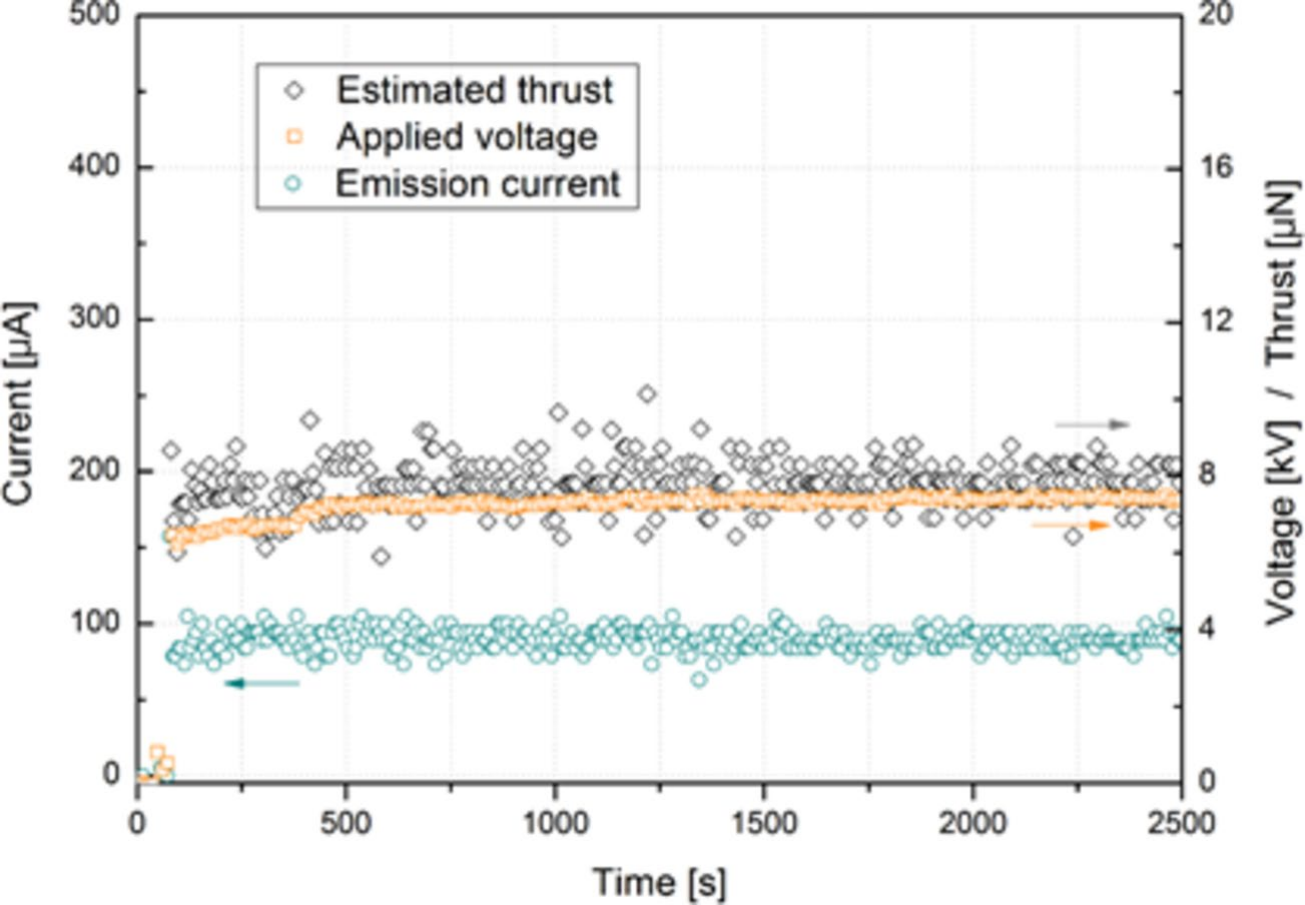
Preliminary test results of the FEEP thruster with emission current, applied voltage, and estimated thrust.

Figure 18 presents the time-resolved emission current, applied voltage, and estimated thrust. During the initial test, a stable emission current of approximately 100 µA was obtained at an average applied voltage of 7037.11 V, corresponding to an average thrust of 7.73 µN. The thrust was estimated from the measured beam current I and applied voltage V using Eq. (3). The achieved emission current level (~ 100 µA) and corresponding thrust scale (~ 8 µN) are in good agreement with previously reported performance of FEEP emitters, including ~ 100 µA and ~ 10 µN for indium emitters^42^, a maximum of ~ 325 µA and 30 µN^43^, and ~ 100 µA and ~ 8 µN for gallium propellant^17^. These comparisons indicate that the estimated performance falls within a physically realistic and expected operational regime.

Although the applied current level is within a typical operating range for FEEP emitters, the present test was limited in duration and should therefore be interpreted as a preliminary functional demonstration rather than a lifetime qualification experiment. The experimental results demonstrate the feasibility of field emission from the fabricated porous tungsten emitter under FEEP operating conditions. The ability to maintain stable emission indicates continuous propellant supply through the porous structure, suggesting that the emitter geometry and internal porosity are functionally effective. While direct thrust measurements would provide further validation, the indirect thrust estimation and emission behaviour indicate that the developed etching process is capable of producing functional emitters for FEEP applications. These findings establish a process-level framework for understanding and controlling porous tungsten etching, providing a basis for future studies linking fabrication conditions with emitter performance.

**Table 3:** Elemental composition obtained from EDS quantitative analysis.

Element	Weight (%)	Atomic (%)
C	1.74	8.26
O	9.74	34.69
Fe	0.13	0.13
Al	1.64	3.47
W	59.81	18.53
K	15.82	23.05
Cr	5.93	6.50
Mn	5.18	5.37

## Conclusion

The present study provides a systematic investigation of electrochemical etching behaviour in porous tungsten for the fabrication of micro-emitters with controlled tip geometry. A comprehensive parametric study demonstrated that conventional electrochemical etching, while effective for solid tungsten, leads to limitations in porosity preservation and the resulting tip morphology when applied to porous materials. In contrast, the incorporation of discharge-assisted etching enabled controlled material removal, allowing the formation of sharp tips with the porous structure preserved. A key finding of this study is that the process window for viable porous emitter fabrication is significantly constrained. Distinct etching regimes were identified, and failure modes were observed outside this narrow voltage range. The resulting tip diameter exhibited a strong dependence on the applied voltage, and voltage-dependent etching behaviour was classified into four distinct regimes (Zones A–D) over an extended voltage range up to 100 V. Except for the highest voltage range (80–100 V, Zone D), the target tip diameter of approximately 5 μm was generally achievable across the investigated conditions. In the low-voltage regime (5–12 V, Zone A), concave tip morphologies were formed despite achieving the target diameter. At intermediate voltages (12–35 V, Zone B), prolonged discharge led to the formation of a dense surface crust, which affected porosity preservation. In the range of 40–60 V (Zone C), convex porous tips with preserved structure were obtained, representing the favourable fabrication window. At higher voltages (80–100 V, Zone D), excessive discharge caused severe melting and loss of porosity, resulting in significant deviation from the target geometry. In the intermediate voltage regime (Zone B), localised melting and the formation of a dense multicomponent crust layer were observed on the surface. EDS analysis showed this crust to be enriched in oxygen and potassium (W 59.8 wt%, O 9.7 wt%, K 15.8 wt%), consistent with a mixture of tungsten oxide species and electrolyte-derived compounds rather than pure metallic redeposition. This layer effectively sealed the underlying porous network, which limited the preservation of porosity. The novelty of this work lies in the systematic mapping of the etching parameter space and the identification of regime-dependent morphological transitions and failure modes in porous tungsten. This establishes a process-level framework for understanding and controlling porous tungsten etching, providing a foundation for future studies linking fabrication conditions with emitter performance.

## Data Availability

All data generated or analysed during this study are included in this published article.

## Abbreviations

e: elementary charge
ɛ_o_: permittivity of free space
η: mass efficiency
f: thrust correction coefficient
g: gravitational acceleration
γ: surface tension
h: distance between extractor and emitter
I: emission current
I_sp_: specific impulse
m: mass of emitted ions from propellant
$\:{\dot{m}}_{ion}\:$: ion mass flow rate
$\:{\dot{m}}_{droplet}\:$: droplet mass flow rate
$\:{\dot{m}}_{total}\:$: total mass flow rate of emitted species
φ: half-angle of the Taylor cone
R: radius of the emitter tip
$\:{\mathrm{r}}_{\mathrm{b}\mathrm{a}\mathrm{s}\mathrm{e}}$: base radius of the Taylor cone
T: generated thrust
V: applied voltage
V_0_: emission onset voltage
